# Acid‐Stable Nitrofuran Triple Therapy With Rabeprazole and Amoxicillin Targeting *Helicobacter pylori* Infection

**DOI:** 10.1002/mco2.71057

**Published:** 2026-10-11

**Authors:** Nagendran Tharmalingam, Kulandaisamy Arulsamy, Shaolei Lu, Sadhna Dhingra, Scott Goodrich, Joseph St Laurent, Gerald Jones, Eleftherios Mylonakis

**Affiliations:** ^1^ Department of Medicine Houston Methodist Research Institute Houston Texas USA; ^2^ Department of Medicine Weill Cornell Medicine New York New York USA; ^3^ Boston Children's Hospital Boston Massachusetts USA; ^4^ Department of Pathology Rhode Island Hospital Warren Alpert Medical School of Brown University Providence Rhode Island USA; ^5^ Department of Pathology Houston Methodist Hospital Houston Texas USA; ^6^ Koda Therapeutics West Bridgewater Massachusetts USA

**Keywords:** antibacterial, C57BL/6J mouse, *H. pylori*, mono therapy, nitrofuran derivatives

## Abstract

*Helicobacter pylori* is an emerging antibiotic‐resistant and carcinogenic gastric pathogen. We developed nitrofuran derivatives with anti‐*H. pylori* activity and found the analog CLI‐19‐009 (2‐nitro‐6,7‐dihydrobenzofuran‐4‐one N‐(cinnamoyl)hydrazone) inhibited *H. pylori* growth at 0.25 mg/L. Notably, CLI‐19‐009 contains a non‐conjugated hydrazone side chain, which may influence nitroreductive activation compared with classical nitrofurans and retain activity under acidic conditions. CLI‐19‐009 exhibited an additive effect with clinical antibiotics and proton pump inhibitors, cleared *H. pylori* on gastric epithelial cells, reduced bacterial motility, and decreased vacuolation. CLI‐19‐009 also showed low cytotoxicity (IC_50_>64 mg/L) and significantly improved survival of *H. pylori*‐infected wax moth larvae compared with untreated controls (*p = *0.0002). Host‐cell RNA sequencing revealed that CLI‐19‐009 treatment downregulated inflammatory genes (IL1B, IL18, IL23A, MAPK1, NFKB1, TNF, and IRAK1) during host–pathogen interactions. Long‐term exposure generated CLI‐19‐009‐resistant clones harboring a single‐nucleotide polymorphism in *rdxA* in both lineages, demonstrated using whole‐genome sequencing; this finding suggests alterations in nitroreductive activation. CLI‐19‐009 showed in vitro synergy with rabeprazole and additive activity with amoxicillin, and these combinations reduced gastric inflammation and colonization compared with untreated mice when tested in a murine infection model (*p* < 0.0001). These findings support further development of CLI‐19‐009 as an anti‐*H. pylori* therapeutic alternative to clarithromycin‐based regimens.

## Graphical abstract

**Overview**: Novel nitrofuran derivatives were developed and evaluated against *Helicobacter pylori* to overcome antibiotic resistance and inflammation.

**Methods and Findings**: In vitro assays using *H. pylori* 60190 and PMSS1 strains demonstrated potent antibacterial activity, reduced bacterial adhesion, and inhibited cytokine release from gastric epithelial cells. RNA sequencing of host‐pathogen interactions revealed significant downregulation of inflammatory (IL1B, IL18, IL23A) and oncogenic (CDK9) gene expression. The compounds showed strong synergism with clinical agents such as amoxicillin and rabeprazole, effectively preventing the emergence of resistant mutants.

**In vivo efficacy**: In a C57BL/6J mouse model, treatment with the lead compound CLI‐19‐009, alone or in triple therapy with amoxicillin and rabeprazole, eradicated gastric infection, reduced inflammation, and exhibited hepatic and renal safety.

**Conclusion**: Nitrofuran‐based triple therapy targets both *H. pylori* pathogenesis and inflammation, offering a promising alternative to resistance‐prone clarithromycin‐based regimens.

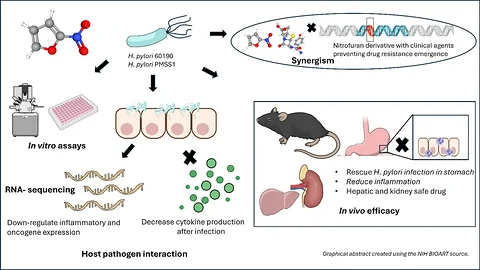

## 1 Introduction

*Helicobacter pylori* is a gram‐negative stomach pathogen capable of causing chronic gastritis, peptic ulcers, gastric mucosa‐associated lymphoid tissue lymphoma, and gastric carcinoma [1]. Antibiotic resistance to *H. pylori* continues to rise globally, necessitating the development of a new class of antimicrobial agents [2]. Several new antibiotics have emerged that show promise in treating multidrug‐resistant strains (MDRS) of *H. pylori*. Rifabutin‐based therapy has achieved high bacterial eradication rates, even in patients for whom other treatments failed [3], and sitafloxacin, a new fluoroquinolone, demonstrated high eradication rates in MDRS cases [4]. Rifaximin, primarily used for gastrointestinal infections, may also be effective against *H. pylori*, including MDRS [5]. Nitazoxanide, an antiparasitic, has shown anti‐*H. pylori* potential alone and in combination with other antibiotics [5], and fidaxomicin, known for treating *Clostridioides difficile* infections, has also demonstrated activity against MDRS of *H. pylori* [5, 6].

Building on this exploration of new and existing antibiotics for treatment against MDRS of *H. pylori*, our study focuses on the development of nitrofuran derivatives with promising antibacterial and anti‐virulence properties. Nitrofurans are fused heteroaromatic compounds with exceptional therapeutic and biological activities [7]. While nitrofuran‐based therapies, including furazolidone, have been explored in treatment against *H. pylori* [8, 9] and have demonstrated efficacy in combination therapy [10, 11], we introduce the development of structurally distinct nitrofuran derivatives incorporating hydrazone‐linked side chains that are not conjugated to the nitro moiety. This in turn, may alter their redox behavior and mechanism of action. This strategy aims to overcome limitations of conventional nitrofuran agents by improving activity under acidic conditions, reducing cytotoxicity, and expanding antibacterial and anti‐virulence properties. Of 16 total analogs screened, we selected four analogs containing a nitrofuran scaffold backbone to investigate their antibacterial and anti‐virulence potential. From these, one analog was further investigated for in vitro and in vivo validation. This study was designed as a tiered translational framework to systematically evaluate the antibacterial, anti‐virulence, mechanistic, and in vivo therapeutic effects of structurally modified nitrofuran derivatives. The workflow progresses from in vitro assessment, anti‐virulence assays, systems‐level bacterial and host transcriptomic and metabolic profiling, resistance evolution, and genomic characterization, to validation in independent in vivo infection models. This stepwise design critically enables cross‐validation of findings across orthogonal biological systems

## 2 Results

### 2.1 Antibacterial Susceptibility and Activity

A screen of nitrofuran analogs (KODA Therapeutics, MA, USA) against the model organism *H. pylori* strain 60190 revealed a range of minimum inhibitory concentration (MIC) values from 0.25 to 4 mg/L (Figure S1A). From these results, we selected four analogs with low MIC values to explore their anti‐*H. pylori* activity. The structure of each of these four analogs is illustrated in Figure 1A. Three of the analogs share a common dihydrobenzofuran backbone, CLI‐19‐008 (2‐nitro‐6,7‐dihydrobenzofuran‐4‐one N‐(ethyl phenylphosphoramidoyl)hydrazone), CLI‐19‐009 (2‐nitro‐6,7‐dihydrobenzofuran‐4‐one N‐(cinnamoyl)hydrazone), and CLI‐03‐303 (2‐nitro‐6,7‐dihydrobenzofuran‐4‐one semicarbazone), and inhibited *H. pylori* growth with an MIC of 0.25 mg/L and a minimum bactericidal concentration (MBC) of 0.25 mg/L. The fourth analog, CLI‐03‐258 (5‐nitro‐2‐furaldehyde N‐(ethyl phenylphosphoramidoyl)hydrazone), contains a furaldehyde backbone and exhibited an MIC of 1.0 mg/L and an MBC of 2.0 mg/L. Together, the selected nitrofuran analogs inhibited *H. pylori* reference strains with different genetic backgrounds, including 60190, NCTC 11637, Tx30a (Cag A negative strain) (Figure 1B), and a panel of clinical *H. pylori* isolates with variable susceptibility profiles to clinical antibiotics (Figure S1B). Notably, several clinical isolates with resistance to clarithromycin and metronidazole remained susceptible to CLI‐19‐009. We also evaluated the acid stability of the nitrofuran derivatives and found that nitrofuran derivatives were more active under acidic conditions, with the MIC decreasing from 0.25 to 0.06 mg/L at pH 5.5 (Figure 1C).

**FIGURE 1:**
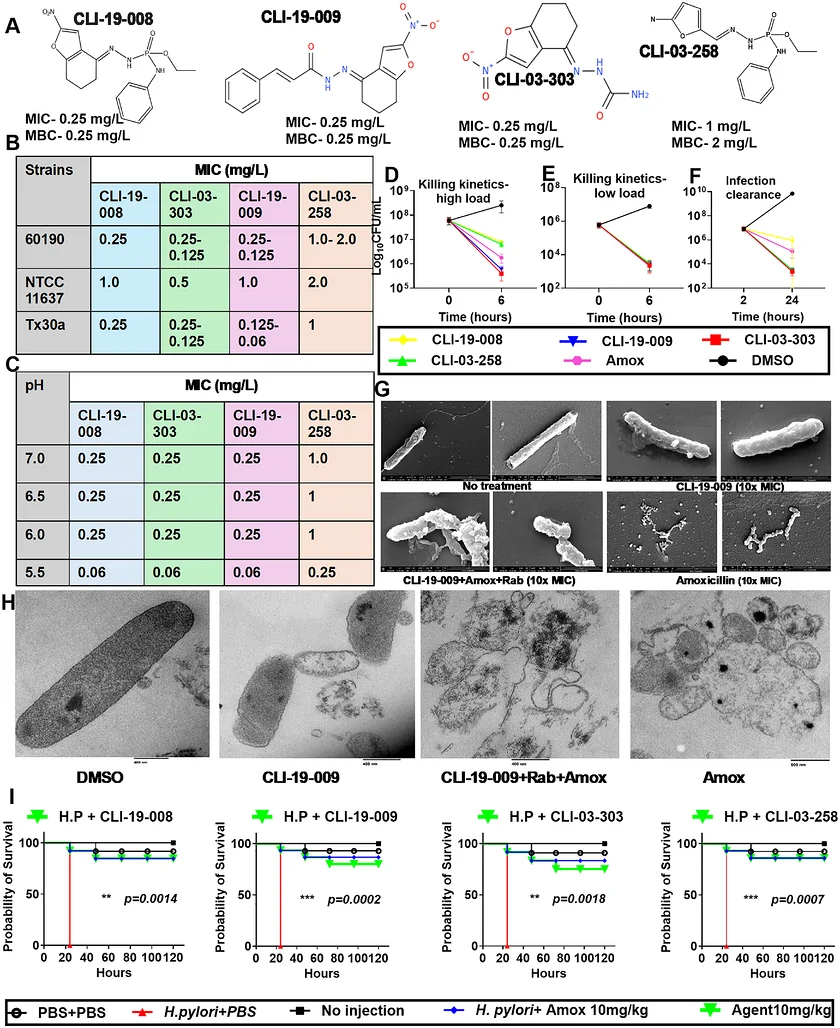
Structure and inhibitory activity of nitrofuran derivatives against *H. pylori*. (A) Structure, MIC, and MBCs of nitrofuran derivatives against *H. pylori* strain 60190. (B–C) Antibacterial activity of nitrofuran derivatives against different strains and conditions. (B) MIC of nitrofuran derivatives against other *H. pylori* reference strains with varying genetic backgrounds based on the EPIYA motif of Cag A and CagA negative strain. (C) MIC of nitrofuran derivatives against *H. pylori* strain 60190 at acidic pH levels ranging from 7 to 5.5 (*n = *3 biologically independent samples). (D–E). Time‐to‐kill assays in which a high dose (D) or low dose (E) of exponentially growing *H. pylori* were treated with nitrofuran derivatives at 4x MIC; amoxicillin or DMSO served as controls. Colony‐forming units (CFU) at different time intervals were plotted. *p*‐value for nitrofuran derivatives or Amox (4x MIC vs untreated) is < 0.01 for high dose and < 0.0001 for low dose; 2‐way‐ANOVA. (F) Infection clearance assay showing the total viable count demonstrated a reduction in total CFU after 20 h of treatment. Graphs (D–E) show data with means ± SD for *n = *3 biologically independent samples. *p*‐value for nitrofuran derivatives or Amox (4x MIC vs untreated) is less than *p* < 0.0001 for high dose; 2‐way‐ANOVA (G–H). Microscopy of *H. pylori* treated with CLI‐19‐009 alone or in triple therapy. Scanning electron microscopy (G) shows the flagellar assembly and cell morphology in *H. pylori* after treatment with CLI‐19‐009 (10x MIC). Representative images from a pool of three independent experiments are shown. (H) Transmission electron microscopy of untreated and CLI‐19‐009 alone or in triple therapy‐treated *H. pylori* shows the morphological changes and cellular fitness was compromised. Representative images from a pool of three independent experiments are shown. (I) In vivo infection assay in *G. mellonella* larvae injected with *H. pylori* 60190 and treatment with CLI‐19‐008, CLI‐19‐009, CLI‐03‐303, or CLI‐03‐258 at 10 mg/kg/larvae expanded the survival of the larvae with *H. pylori* infection. Survival was monitored for *n = *16 larvae. The representative figure shows data from three independent repeats. Survival analysis was performed using the Log‐rank (Mantel–Cox) test (conservative). Survival curves were compared across groups and showed a statistically significant difference (*p* < 0.01).

Cytotoxicity assays using multiple cell lines demonstrated that the four compounds exhibited no toxicity up to 64 mg/L (Figure S1C). Time‐to‐kill assays revealed that all four compounds inhibited *H. pylori* growth at 4× MIC in the very high bacterial‐load experimental condition (1.0 OD_600_) compared to dimethyl sulfoxide (DMSO) controls (Figure 1D). All four compounds also exhibited bactericidal activity at a 3‐log_10_ colony‐forming unit (CFU) reduction compared to the DMSO control in the low‐load condition (0.5 OD_600_) (Figure 1E). We next evaluated the ability of the nitrofuran compounds to clear *H. pylori* colonization in human gastric epithelial (AGS) cells and observed that the administered concentration of dihydronitrofuran derivatives effectively cleared colonization with a resultant 4‐log_10_ reduction in CFU counts (Figure 1F).

Scanning electron microscopy (SEM) demonstrated that the untreated cells exhibited typical helical rod‐shaped morphology with smooth surfaces and intact flagellar structures. In contrast, CLI‐19‐009‐treated cells displayed significant morphological alterations, including reduced cell length, surface irregularities, and disruption of flagellar architecture (Figure 1G). Structural changes were more pronounced under triple therapy conditions (CLI‐19‐009 + rabeprazole+amoxicillin), where extensive deformation, fragmentation, and loss of cellular integrity were observed. Transmission electron microscopy (TEM) confirmed that the untreated cells displayed intact morphology with well‐defined membranes and uniform cytoplasmic content. In contrast, cells treated with CLI‐19‐009 demonstrated reduced gross cellular length and pronounced intracellular alterations, including cytoplasmic condensation and disruption of membrane integrity (Figure 1H).

We next evaluated the efficacy of nitrofuran derivatives against an *H. pylori* infection model using wax‐moth larvae as an invertebrate model host. With treatment, we observed a survival rate up to 70%, representing a significant improvement compared to the no‐treatment group (CLI‐19‐008, *p* < 0.0014; CLI‐19‐009, *p* < 0.0002; CLI‐03‐303, *p* < 0.0018; CLI‐03‐258, *p* < 0.0007) (Figure 1I). The clarithromycin‐injected group served as the positive control, achieving up to 80% survival after five days.

### 2.2 Chemical Stability and Anti‐Virulence Effect of CLI‐19‐009

We evaluated the chemical stability of the test compounds under high‐acidic conditions and found that the four nitrofuran derivatives retained antibacterial activity at acidic pH (Figure 1C). We further assessed the structural stability and antibacterial activity of the compounds following exposure to pH 2.0 using standard assays and flow‐injection analysis‐mass spectrometry (FIA‐MS), respectively. Antibacterial assays at pH 2.0 pretreatment showed no change in MICs. Comparative spectral analysis demonstrated that the major molecular ion peaks remained structurally intact after acid treatment with no significant degradation of products observed (Figure 2A), supporting acid stability.

**FIGURE 2:**
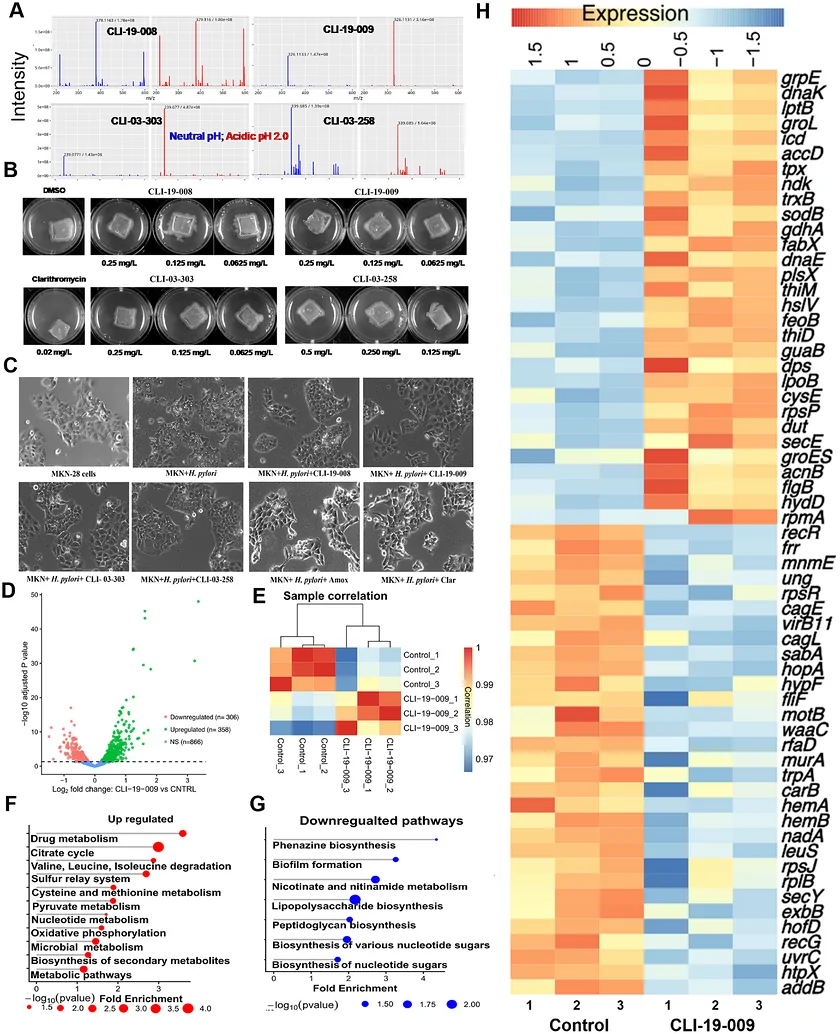
Chemical stability and antivirulence effect of CLI‐19‐009. (A) Acid stability of nitrofuran derivatives. The compounds were treated with preadjusted acetonitrile (pH 2.0) and analyzed. An intact structure demonstrated that the compounds are stable at acidic pH. (B) Motility assay in which agar‐grown *H. pylori* in motility agar (0.4% agar) with test compounds reduced the migration of bacteria through the agar from the inoculation point to the plate's periphery was observed. (C) Light microscopy (40x) was used to examine vacuolation caused by *H. pylori* VacA toxin in gastric epithelial cells (reduced by the test compounds treatment). Scale bar—Low power magnification. (D–H). RNA‐seq analysis of CLI‐19‐009 treated *H. pylori* (*n = *3). (D) Volcano plot of differentially expressed genes between CLI‐19‐009‐treated and control *H. pylori* samples. Differential expression analysis was performed using DESeq2 negative binomial generalized linear models with Wald significance testing and Benjamini–Hochberg false discovery rate (FDR) correction. (E) Pearson correlation heatmap of variance‐stabilized transcriptomic profiles showing strong replicate reproducibility and clear separation between control and CLI‐19‐009‐treated samples. (F–G) KEGG pathway enrichment analysis of significantly upregulated genes (F) and downregulated genes (G) in CLI‐19‐009‐treated *H. pylori*. Dot size indicates enrichment significance (−log10 *p*‐value), and the x‐axis represents fold enrichment. Pathway enrichment was performed using hypergeometric testing implemented in the enricher function of clusterProfiler. (H) Heatmap showing the expression patterns of selected differentially expressed genes across biological replicates. Expression values represent row‐wise scaled variance‐stabilized transcript levels.

We next evaluated the anti‐virulence potential of CLI‐19‐009 using motility assays. Treatment with CLI‐19‐009 at the 1× MIC or sub‐MIC concentrations significantly reduced bacterial motility (Figure 2B). In addition, we evaluated *H. pylori*‐induced vacuolation in MKN‐28 cells. Treatment with the test compounds at 4× MIC significantly reduced *H. pylori*‐induced vacuolation compared with the untreated infected control (Figure 2C). Clarithromycin and amoxicillin served as positive control antibiotics. To confirm the anti‐virulence properties of CLI‐19‐009, we also ran RNA sequencing analyses of *H. pylori* cells treated with a sub‐MIC of CLI‐19‐009; we found a broad transcriptional stress response characterized by activation of oxidative stress defense, protein quality control, membrane adaptation, and metabolic survival pathways, alongside suppression of virulence, motility, DNA repair, and biosynthetic functions. Volcano plot analysis demonstrated substantial transcriptional remodeling, with 358 upregulated and 306 downregulated genes in CLI‐19‐009‐treated *H. pylori* cells (Figure 2D).

Correlation analysis indicated high reproducibility among biological replicates and clear separation between control and CLI‐19‐009‐treated groups (Figure 2E), indicating a robust transcriptomic response to CLI‐19‐009 treatment. KEGG pathway enrichment analysis of upregulated genes revealed pathways associated with drug metabolism, citrate cycle (TCA cycle), oxidative phosphorylation, sulfur relay system, pyruvate metabolism, and cysteine and methionine metabolism (Figure 2F). These findings suggest activation of metabolic‐ and redox‐associated adaptive responses following CLI‐19‐009 treatment. In contrast, downregulated genes were significantly enriched in pathways related to biofilm formation, lipopolysaccharide biosynthesis, peptidoglycan biosynthesis, and nucleotide sugar metabolism (Figure 2G), indicating coordinated downregulation of pathways associated with virulence, motility, and cell envelope functions.

The heat map in Figure 2H illustrates the up‐ and down‐regulated genes in *H. pylori* following CLI‐19‐009 exposure. The top upregulated genes included molecular chaperones and heat‐shock regulators including *grpE, dnaK, groL, groES*, and *hslV*, indicating induction of proteotoxic stress. Oxidative stress defense genes such as *tpx, trxB, sodB*, and *dps* were also elevated, suggesting increased reactive oxygen stress. In addition, genes involved in membrane and lipid homeostasis (*accD, fabX, plsX, lptB, lpoB*), central metabolism (*icd, gdhA, acnB, guaB)*, iron transport (*feoB*), nucleotide metabolism (*ndk, dut*), and secretion‐associated pathways (*secE*) were upregulated, consistent with a bacterial adaptive survival response under antimicrobial pressure.

In contrast, CLI‐19‐009 strongly suppressed several major virulence‐associated pathways in *H. pylori*. Specifically, components of the cag pathogenicity island, including *cagE, virB11*, and *cagL*, were significantly downregulated, indicating impaired Type IV secretion system activity and reduced virulence potential. Adhesion‐associated outer membrane genes *sabA* and *hopA* were also downregulated, suggesting diminished colonization capacity. Genes associated with urease maturation and acid adaptation (*ureF, hypF*) were downregulated, potentially compromising gastric survival. Furthermore, motility‐associated genes (*fliF, motB*) were downregulated, indicating impaired flagellar assembly and motility.

CLI‐19‐009 treatment additionally disrupted envelope biogenesis and DNA maintenance pathways. Downregulation of *waaC, rfaD*, and *murA* suggests defects in lipopolysaccharide and peptidoglycan biosynthesis, potentially weakening membrane integrity. Multiple genes involved in DNA replication and repair, including *recR, recG, uvrC*, *ung*, and *addB*, were suppressed, indicating compromised genome maintenance and stress recovery mechanisms. Translational machinery components such as *rpsR, rpsJ, rplB, frr*, and *leuS* were also downregulated, reflecting inhibition of protein synthesis. Collectively, these transcriptional alterations demonstrated that CLI‐19‐009 exerts a multifaceted antibacterial effect by simultaneously inducing cellular stress responses while suppressing virulence, motility, envelope maintenance, and essential survival pathways in *H. pylori*.

Finally, we utilized untargeted metabolomics to better understand the role of CLI‐19‐009 against *H. pylori*. We found that it perturbed amino acid, nucleotide, and most importantly, nitrogen metabolism (Figure S2). We also examined the test compounds that may play a role in the bacterial membrane. None of the test compounds, except CLI‐03‐303 and CLI‐19‐009 at high concentrations, affected membrane polarization, membrane potential, or membrane integrity (CCCP and Triton‐X were used as positive controls) (Figure S3). However, we did observe a mild decrease in membrane potential following treatment with CLI‐03‐303 and CLI‐19‐009 at a high concentration (32 mg/L).

### 2.3 Role of CLI‐19‐009 on Host–Pathogen Interaction

We used an established gastric infection model with C57BL/6J mice and the mouse‐adapted *H. pylori* strain PMSS1 to test the efficacy of orally administered nitrofurans in mono, dual, or triple therapy compared to a standard antibiotic control group. After three weeks of infection, oral monotherapy was initiated at a dose of 20 mg/kg for a 14‐day treatment period [12]. Dose selection was guided by an integrated assessment of in vitro antimicrobial potency, bactericidal activity, therapeutic index, and preliminary in vivo efficacy data. CLI‐19‐009 demonstrated low MIC and MBC values against *H. pylori* (0.25 mg/L), including bactericidal activity in time‐kill assays under both low‐ and high‐inoculum conditions, while maintaining activity under acidic pH conditions relevant to the gastric environment. Mammalian cytotoxicity assays demonstrated minimal toxicity at concentrations substantially exceeding the active antimicrobial range, supporting a favorable selectivity profile. In parallel, preliminary efficacy and tolerability studies in the wax moth (*Galleria mellonella*) infection model demonstrated significant survival benefit at 10 mg/kg without observable toxicity (Figure 1I). Collectively, these findings supported escalation to 20 mg/kg in the murine model to account for the greater pharmacokinetic and physiological complexity of mammalian hosts, consistent with empirical dose‐escalation strategies commonly used in preclinical antimicrobial efficacy studies. Analysis of H&E‐stained stomach tissue samples using the Sydney System [13, 14] showed that the CLI‐19‐009‐treated group had a significantly lower inflammation score compared to the untreated *H. pylori*‐infected group (0.75 vs. 1.5, *p* < 0.001; Figure 3A). The antibiotic control group, which received amoxicillin (1 mg/kg), clarithromycin (10 mg/kg), and omeprazole (10 mg/kg), had an inflammation score of 0.5. Immunohistochemical staining with polyclonal antibodies to visualize *H. pylori* demonstrated bacterial clearance from the infected area (Figure 3B). The nitrofuran derivatives did not affect blood cell counts (Figure 3C), cause in vivo cytotoxicity (alanine transferase/blood urea nitrogen; Figure 3D), or lead to a change in body weight (Figure 3E).

**FIGURE 3:**
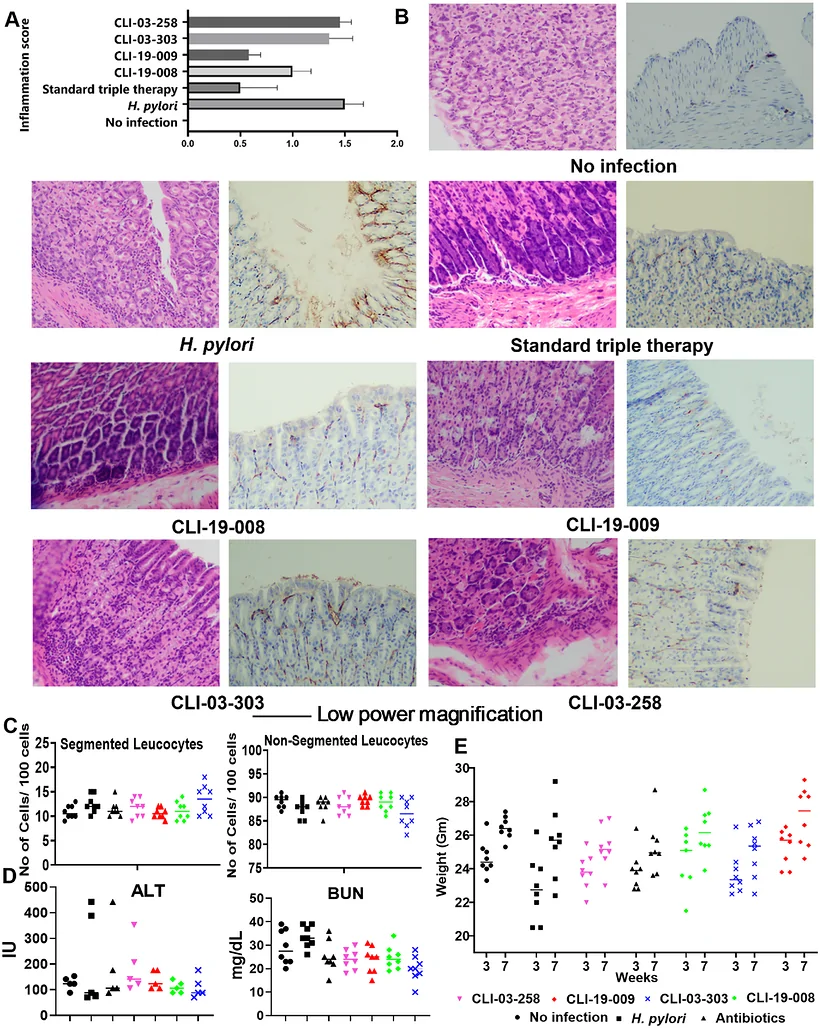
In vivo efficacy of nitrofuran derivatives against *H. pylori* infection. (A) Inflammation score of H&E‐stained stomach sections from *H. pylori*‐infected C57BL/6J mice. Data are presented as means ± SD (*n = *8). Multiple comparisons were performed using Dunnett's multiple comparisons test. One way ANOVA indicated improved outcomes in these treatment groups (*p* < 0.0001) compared to *H. pylori* infection. (B) *H. pylori* strain PMSS1 administered C57BL/6J mice were treated with test compounds (20 mg/kg) show, CLI‐19‐009 treatment cleared *H. pylori*. Scale bar—Low power magnification. (C) Differential blood count was performed to assess the immune response during infection, and the treatment showed no significant changes. (D) Kidney and liver function tests show no cytotoxic effects of the test compounds on vital organs. (E) Analysis of body weights measured before and after oral drug therapy shows no significant changes between groups.

We next evaluated CLI‐19‐009 in dual therapy with proton pump inhibitors (PPIs); among the four selected compounds, CLI‐19‐009 was chosen as the best candidate from the in vivo results described above. To evaluate the combinatorial activity of CLI‐19‐009 in vivo with other clinical drugs used to treat *H. pylori*, we first combined CLI‐19‐009 at three different concentrations (10, 20, or 30 mg/kg) in dual therapy with the PPI omeprazole (10 mg/kg). At 10 mg/kg, CLI‐19‐009 reduced inflammation in both the presence and absence of omeprazole (Figure S4A,B) and did not cause any significant changes in liver or kidney enzymes or body weight (Figure S5A,B).

To better understand the host's immune response to *H. pylori* infection, we used a mammalian cell‐*H. pylori* infection model to investigate the gene expression profiles of gastric epithelial cells (MKN‐28) treated in the presence of *H. pylori* infection (Figure 4). Principal Component Analysis (PCA) demonstrated distinct clustering of RNA‐sequencing samples, including MKN+HP (*H. pylori*‐infected), MKN+HP+CLI‐19‐009, and uninfected MKN control samples, indicating treatment‐driven transcriptomic shifts (Figure 4A). Transformed counts showing the overall distribution of gene expression revealed the treated samples were significantly different from the untreated infection control (Figure 4B **)**. The heat map of *H. pylori*‐infected MKN‐28 cells illustrates upregulation of inflammation‐associated genes, including *CCL5, IL1B, IL18, IL23A*, MAPK1 (MAPK signaling protein), NFKB1 (transcription factor for inflammation and other signal transduction protein), *TNF* (tumor necrosis protein involved in inflammatory cytokine production), and *IRAK1* (encodes the interleukin‐1 receptor‐associated kinase 1 (IRAK‐1) crucial for regulating inflammatory response) were downregulated in *H. pylori*‐infected cells treated with CLI‐19‐009 (Figure 4C). In addition, we found several genes involved in cellular regulation which were downregulated in *H. pylori* infection, including *TGFB1* (producing transforming growth factor beta‐1 (TGFβ‐1); involved in regulating cell growth), *ELF4* (crucial for innate immunity, NK cell development, and inflammation regulation), and *HOXA13* (binds to specific regions of DNA; helps control the activity of other genes). Notably, the downregulation of these genes was reversed following CLI‐19‐009 treatment. Gene enrichment studies based on KEGG pathway analysis show the selected inflammation‐ and cancer‐associated genes that were downregulated by CLI‐19‐009 treatment (Figure 4D,E), mediated the reduction in bacterial burden.

**FIGURE 4:**
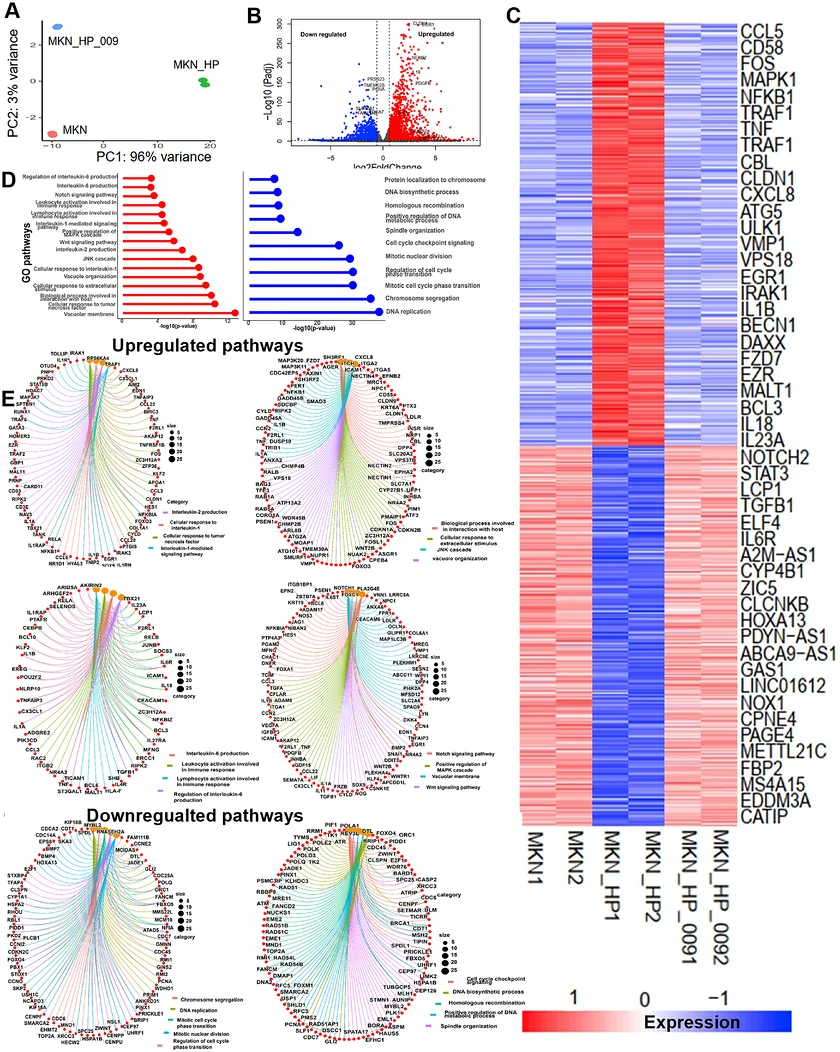
Transcriptomic analysis of differential gene expression and pathway enrichment in *H. pylori* Infection. MKN‐28 cells were infected with *H. pylori* 60190 for 2 h and were then incubated in the presence or absence of CLI‐19‐009 for 22 h. RNA was isolated, sequenced, and processed using DESeq2 pipeline for differential expression analysis. (A) Principal component analysis (PCA) plot demonstrating distinct clustering of RNA‐seq samples, including MKN+HP (*H. pylori*‐infected), MKN+HP+CLI‐19‐009, and uninfected MKN control samples, indicating treatment‐driven transcriptomic shifts. PCA was performed using variance‐stabilized expression values generated by DESeq2. (B) Volcano plots displaying differentially expressed genes (DEGs) between MKN_HP and each of the other groups (MKN+HP+CLI‐19‐009). Differential expression analysis was performed using DESeq2 negative binomial generalized linear models with Wald significance testing and Benjamini–Hochberg false discovery rate (FDR) correction. Significantly upregulated and downregulated genes are highlighted. (C) A heatmap was generated to visualize the top 40 upregulated and downregulated genes. Expression values represent row‐wise scaled variance‐stabilized transcript levels generated by DESeq2. (D) Lollipop plot showing significantly enriched biological pathways associated with differentially expressed genes across MKN, MKN+HP, and MKN+HP+CLI‐19‐009 transcriptomic comparisons. (E) Functional enrichment analysis was performed using Gene Ontology over‐representation analysis implemented in the enrichGO function of clusterProfiler based on hypergeometric testing. Pathway significance was determined using *p* values < 0.05.

### 2.4 Mutational Frequency Studies

Probing the combinatorial activity of nitrofuran with clinical antibiotics, we found the three dihydrobenzofuran derivates exhibited an additive effect (fractional inhibitory concentration index (FICI), ranging from 0.75 to 1.0) with amoxicillin, clarithromycin, and the PPIs omeprazole and pantoprazole (Figure 5A). Notably, none of the compounds were antagonistic with other clinical drugs in combination. We performed a mutation frequency assay to determine whether drug resistance emerged during the continuous passage of *H. pylori* in the presence of two‐fold serial dilutions of CLI‐19‐009. We observed the emergence of *H. pylori* resistance on the 15th day of continuous exposure, with a two‐fold increase in MIC. After passaging the resistant *H. pylori* for up to 33 days, the MIC of CLI‐19‐009 increased to >16 mg/L (Figure 5B).

**FIGURE 5:**
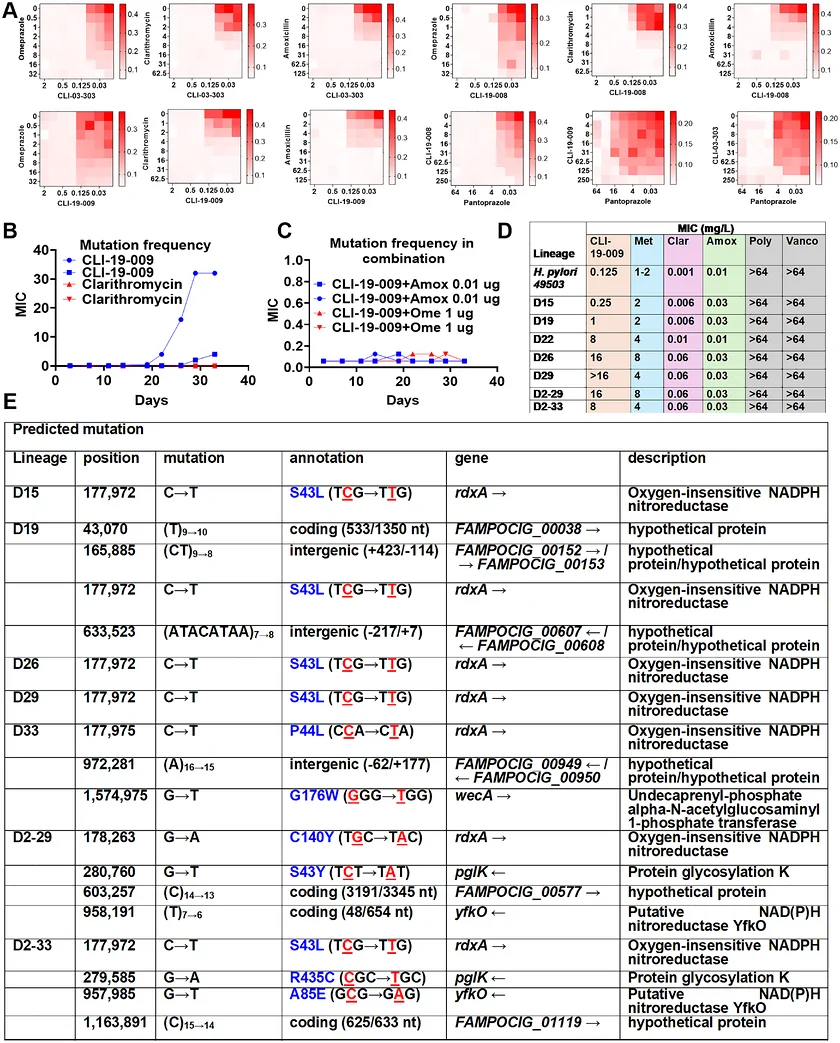
Genomic insights of *H. pylori* against CLI‐19‐009. (A) Combinatorial activity of nitrofuran derivatives. Nitrofuran derivatives were tested with clinical agents using checkerboard assays. FICI analysis demonstrated additive interactions between the nitrofuran derivatives and PPIs (omeprazole or pantoprazole) or clinical antibiotics (amoxicillin or clarithromycin), with FICI values ranging from 0.75 to 1.0. (B) Mutational frequency assay in which two independent *H. pylori* lineages were exposed to two‐fold serially diluted CLI‐19‐009 for 8 passages, with treatments every 3–4 days and generated resistant clones; MICs were plotted. (C) Mutational frequency assay in which *H. pylori* was treated with CLI‐19‐009 in combination with either amoxicillin or omeprazole for 8 passages did not generate resistant clones. (D) The whole genome of two independent *H. pylori* CLI‐19‐009‐resistant clones was sequenced that showing the resistant mutant genes. (E) The MICs of CLI‐19‐009‐resistant clones were measured using the broth microdilution method against a range of clinical antibiotics. CLI‐19‐009‐resistant clones exhibited an increased MICs to metronidazole (MIC: 1–2 to 4 mg/L) and clarithromycin (MIC: 0.001–0.06 mg/L) compared with the wild‐type strain.

We then mimicked the clinical treatment strategy of combination therapy and evaluated whether the combination of test drugs with a PPI or a clinical antibiotic prevents the emergence of drug resistance. No detectable increase in MIC was observed when CLI‐19‐009 was combined with a minimal concentration of amoxicillin (0.01 mg/L) or omeprazole (1 mg/L) (Figure 5C), indicating suppression of resistance emergence under the tested broth microdilution serial passage conditions. To further understand *H. pylori* resistance, we performed whole‐genome sequencing on the CLI‐19‐009‐resistant clones. We identified single nucleotide polymorphisms (SNPs) in *rdxA* (oxygen‐insensitive NAD(P)H nitroreductase) in both lineages and *wecA* (UDP‐N‐acetylglucosamine:undecaprenyl‐phosphate N‐acetylglucosaminyl‐1‐phosphate transferase) in lineage 1 and *yfkO*, (putative NAD(P)H nitroreductase) in lineage 2 (Figure 5D). The MIC of metronidazole increased from 1 to 2 mg/L in the wild‐type strain to 4 mg/L in the CLI‐19‐009‐resistant clone, while the MIC of clarithromycin increased from 0.001 mg/L in the wild‐type strain to 0.06 mg/L in the resistant clone (Figure 5E). However, these changes do not represent phenotypic resistance, as the MIC values remain below the established resistance breakpoints.

### 2.5 In Vivo Efficacy of Nitrofuran Derivatives in Mono or Dual or Triple Therapy

Finally, we tested the in vivo efficacy of a triple therapy regimen combining CLI‐19‐009 with two clinical antibiotics and PPIs. We used the FICI of CLI‐19‐009 with amoxicillin, clarithromycin, omeprazole, and pantoprazole to guide dose selection; CLI‐19‐009 exerted additive effects with omeprazole and amoxicillin (Figure 5A). When CLI‐19‐009 was tested in both mono and dual therapy at 10 mg/kg, it significantly reduced *H. pylori*‐induced inflammation (*p* < 0.0001) and cleared *H. pylori* colonization (Figure S4). No adverse effects were identified in total blood count, including red blood cell and white blood cell counts, or body weight (Figure S5).

Replacing clarithromycin, we investigated CLI‐19‐009 in triple therapy with amoxicillin and omeprazole. Gastric inflammation in mice challenged with CLI‐19‐009 either as a monotherapy or in combination with amoxicillin (1 mg/kg) and omeprazole (10 mg/kg) demonstrated a reduction in inflammation at CLI‐19‐009 10 mg/kg (Figure 6A,B). We further confirmed that CLI‐19‐009 did not show any adverse effects in mono or triple therapy (Figure 6C,D). The standard triple therapy group cleared *H. pylori* colonization compared to the untreated control (clarithromycin + amoxicillin + omeprazole; *p* < 0.0001), reduced inflammation, and served as a positive control for other treatment groups. Amoxicillin (1 mg/kg) + omeprazole (10 mg/kg) did not reduce inflammation (*p = *0.8071) as dual therapy or in combination with CLI‐19‐009 at 1 mg/kg (*p = *0.9996) or 5 mg/kg (*p = *0.9416).

**FIGURE 6:**
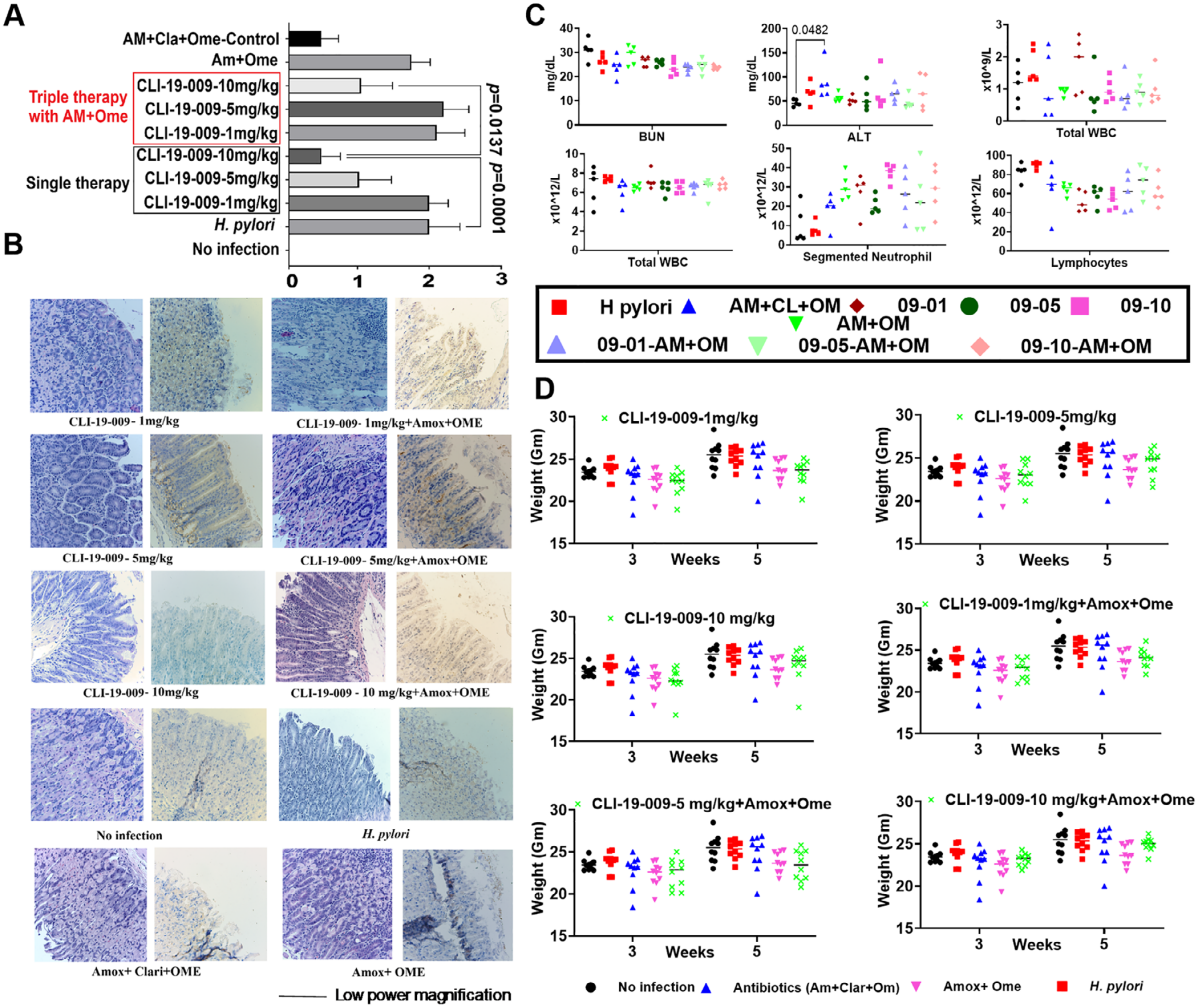
In vivo efficacy of CLI‐19‐009 in mono and triple therapy. (A) *H. pylori* strain PMSS1 administered C57BL/6J mice were treated with CLI‐19‐009 at doses of 1, 5, or 10 mg/kg either in the presence or absence of Ome+Amox dual therapy. Data are presented as means ± SD (*n = *10). Significant differences were observed between *H. pylori* and no infection, Amox+Clari+Ome, 009‐5 mg/kg, 009–10 mg/kg, and 009–10 mg/kg+Amox+Ome groups (*p*<0.0001; one‐way ANOVA), indicating improved outcomes in these treatment groups compared to *H. pylori* infection. (B) Representative images of stomach sections from experimental groups stained with H&E and polyclonal *H. pylori* antibody to visualize bacterial colonization and inflammation are shown. Scale bar—Low power magnification. (C) Whole blood was collected from mice treated with CLI‐19‐009 either in the presence or absence of PPI and amoxicillin (PPI+Amox) show the effects of the treatment on blood parameters. (D) Analysis of body weights measured before and after triple therapy shows no significant changes between groups.

Because CLI‐19‐009 showed only an additive interaction with omeprazole (FICI ranging from 0.75 to 1.0; Figure 5A) but synergy with rabeprazole (FICI < 0.5; Figures 7A and S6–S7), we next replaced omeprazole with rabeprazole to determine whether the stronger in vitro interaction would improve in vivo efficacy. We administered CLI‐19‐009 (5 or 10 mg/kg) in combination with amoxicillin (1 mg/kg) and rabeprazole (10 mg/kg), using a formulation of 1:10 ethanol to corn oil. After 14 days of treatment, CLI‐19‐009 (10 mg/kg) in triple therapy with amoxicillin (1 mg/kg) and rabeprazole (10 mg/kg) effectively reduced (or ameliorated) *H. pylori*‐induced inflammation (Figure 7B) and cleared *H. pylori* colonization (Figure 7C) with no change in body weight (Figure 7D). By replacing resistance‐prone antibiotic clarithromycin, CLI‐19‐009 in triple therapy reduced inflammation similar to the standard of care [15].

**FIGURE 7:**
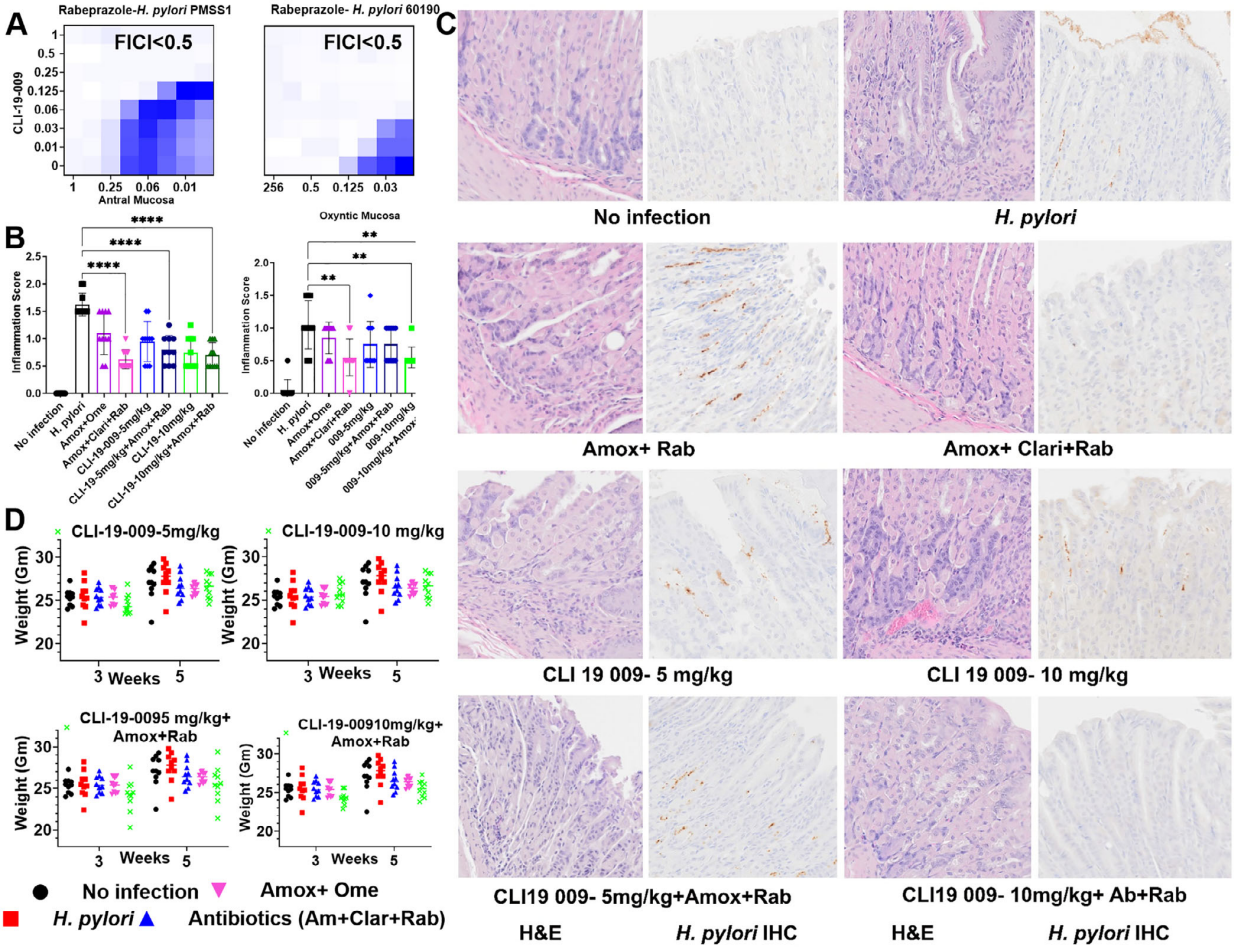
In vivo efficacy of CLI‐19‐009 against *H. pylori* in triple therapy. (A) The combination of CLI‐19‐009 with rabeprazole showing synergy (FICI<0.5). B‐D. C57BL/6J gastric *H. pylori*‐infection model. (B) H&E‐stained stomach sections from *H. pylori*‐infected C57BL/6J mice were analyzed for inflammation. Multiple comparisons were performed using Tukey's multiple comparisons test. All treatment groups showed statistically significant differences when compared with *H. pylori* infection (*p* < 0.0001). Data are presented as means ± SD (*n = *10). (C) *H. pylori* strain PMSS1 was administered orally to C57BL/6J mice, followed by treatment with CLI‐19‐009 at 5 or 10 mg/kg either alone or in combination with rabeprazole and amoxicillin show reduced *H. pylori* infection. Representative images of stomach sections from experimental groups stained with H&E and polyclonal *H. pylori* antibody to visualize bacterial colonization and inflammatory response are shown. Scale bar—Low power magnification. (D) Weight changes were monitored throughout the in vivo efficacy study to assess the impact of CLI‐19‐009 treatment in combination with rabeprazole and amoxicillin on *H. pylori* infection.

## 3 Discussion

The nitrofuran analog CLI‐19‐009 is a hydrazone derived from the condensation of 2‐nitro‐6,7‐dihydrobenzofuran‐4‐one and cinnamic acid hydrazide. Notably, cinnamaldehyde, an α,β‐unsaturated aldehyde [16] structurally related to cinnamic acid, exhibits anti‐*H. pylori* activity and retains its antibacterial activity under acidic conditions [17]. The antibacterial activity of the nitrofuran derivatives was validated against a broader panel of clinical *H. pylori* isolates with heterogeneous antibiotic resistance profiles, supporting the broader applicability of the scaffold beyond laboratory reference strains.

Medications to treat *H. pylori* are delivered orally and must withstand the acidic environment of the stomach. The observed acid stability of CLI‐19‐009 aligns with the findings of Kawasaki et al. [18], who reported acid‐stable nitrofuran derivatives. Notably, CLI‐19‐009 differs from classical nitrofurans in that its hydrazone side chain is not conjugated to the nitro group, a structural feature predicted to alter its redox behavior and nitroreductive activation. This positions the scaffold as a multifunctional anti‐infective platform that influences both pathogen viability and host–pathogen interaction dynamics. In this context, the present work integrates chemical modification of a structurally distinct nitrofuran hydrazone scaffold with antibacterial activity, anti‐virulence phenotyping, resistance evolution analyses, and host‐response modulation within a unified experimental framework.

In addition to its anti‐*H. pylori* activity, CLI‐19‐009 hindered motility and vacuolation, consistent with other studies demonstrating that nitrofuran and cinnamaldehyde scaffold derivatives exert anti‐virulence activity [19, 20, 21]. In addition, CLI‐19‐009 downregulated inflammatory‐response genes associated with *H. pylori* infection [22, 23]. These results indicate that CLI‐19‐009 influenced reduced expression of inflammatory‐response genes, consistent with previous studies of nitrofuran derivatives [24, 25]. Importantly, this work extends beyond conventional nitrofuran optimization by demonstrating that structurally modified hydrazone‐linked nitrofuran derivatives exhibit coordinated antibacterial and anti‐virulence activities while also modulating host inflammatory responses during infection.

The resistant clones carried SNP in *rdxA *(encoding an oxygen‐insensitive NAD(P)H nitroreductase) [26] in both lineages. Because *rdxA* is the principal enzyme that reductively activates the nitroimidazole prodrug metronidazole, the *rdxA *mutation suggests that CLI‐19‐009 may share a nitroreductase‐dependent activation step with metronidazole. Loss of this activating capacity could reduce intracellular generation of reactive nitro intermediates and thereby confer resistance. Once metronidazole is activated, its reduced nitro‐radical intermediates damage DNA [27, 28]. In addition, we observed SNP in *yfkO* (a putative NAD(P)H nitroreductase) in Lineage 2, which is believed to play a role similar to that of *rdxA*. However, the specific function of this gene remains unknown [29]. Distinct from this activation pathway, the *wecA* (UDP‐N‐acetylglucosamine:undecaprenyl‐phosphate N‐acetylglucosaminyl‐1‐phosphate transferase)mutation, together with RNA‐seq evidence of downregulation of genes involved in lipopolysaccharide and peptidoglycan biosynthesis (*waaC, rfaD*, and *murA*), points to a separate effect on cell‐envelope integrity. CLI‐19‐009 resistance therefore appears to involve two distinct components: altered nitroreductive activation and adaptation to cell‐envelope.

Metronidazole resistance is primarily linked to *rdxA* mutations [26], whereas clarithromycin resistance is driven by 23S rRNA substitutions [30]. The presence of *rdxA* and cell envelopes and the associated mutations in resistance clones suggest a combined target‐specific and membrane‐adaptive resistance response. These findings indicate that CLI‐19‐009 imposes strong selective pressure that promotes coordinated multidrug adaptation rather than single‐pathway resistance. Our finding that CLI‐19‐009 combined in dual therapy with either an antibiotic or a PPI shows no detectable increase in MIC under the serial‐passage conditions tested in *H. pylori* is encouraging for drug development. The ability of CLI‐19‐009 in triple therapy with amoxicillin and rabeprazole to reduce gastric inflammation caused by *H. pylori* supports the observation that hybrid therapy of rabeprazole with amoxicillin and clarithromycin achieves high *H. pylori* eradication rates of up to 90% [31].

This study contains several notable limitations. Although multi‐omics, genomic, and phenotypic analyses were integrated to identify potential molecular targets and support CLI‐19‐009's mechanism of action, direct drug–target interactions and the enzymatic pathways responsible for compound activation remain to be experimentally validated. The transcriptomic and genomic findings in this study provide a foundation for these future mechanistic studies. Despite the observed increase in clarithromycin MIC, no mutations were detected in the 23S rRNA gene. Consequently, the mechanism underlying the reduced clarithromycin susceptibility remains unresolved, and any inference of cross‐resistance should be interpreted with caution. Further genetic and functional studies are needed to elucidate the basis of this phenotype. In addition, formal pharmacokinetic/pharmacodynamic analyses were not performed and will be essential to optimize dosing strategies and define exposure–response relationships for clinical translation.

This study establishes the nitrofuran‐based hydrazone scaffold as a promising anti‐*H. pylori* therapeutic platform, identifying CLI‐19‐009 as a lead compound hallmarked by acid stability and strong anti‐virulence effects. Mechanistically, CLI‐19‐009 activity appeared to involve more than single‐target effects characterized by disruption of bacterial metabolic networks, particularly nitrogen and nucleotide metabolism. This finding was supported by whole‐genome sequencing of resistant mutant clones; observation of suppressed virulence factors including motility, and VacA‐mediated vacuolation; and the concurrent attenuation of host inflammatory signaling pathways demonstrated through RNA‐sequencing analyses. In a murine infection model, *H. pylori*‐mediated inflammation was reduced by triple therapy consisting of CLI‐19‐009, amoxicillin, and rabeprazole, compared to a standard regimen. These findings highlight the promising therapeutic capacity of CLI‐19‐009 and related nitrofuran compounds as a meaningful advancement in *H. pylori* therapeutics by expanding the mechanistic and structural diversity of nitrofuran‐based agents, targeting both bacterial viability and virulence‐associated pathways, and demonstrating in vivo efficacy in combination with clinically used agents.

## 4 Methods

### 4.1 Test Compounds and Strains

Four test compounds, CLI‐19‐008, CLI‐19‐009, CLI‐03‐303, and CLI‐03‐258, as well as *H. pylori* strains 60190, NCTC 11637, Tx30a [32] *H. pylori* SS1, and PMSS1 [33], were used in this study. Further details of test compounds and bacterial and culture methods can be found in Supplementary Information.

### 4.2 Antibacterial and Anti‐Virulence Assays

The antibacterial and anti‐virulence characteristics of CLI‐19‐008, CLI‐19‐009, CLI‐03‐303, and CLI‐03‐258 were evaluated using broth microdilution assay [34], time to kill assay [34], motility assay [32], and vacuolation assay. Detailed assay procedures can be found in the Supplementary Information.

### 4.3 Infection Clearance Assay

The MKN‐28 cell line was used to examine treatment‐induced infection clearance [32]. The detailed assay procedure can be found in the Supplementary Information.

### 4.4 Electron Microscopy

*H. pylori* strain 60190 was cultured on *Brucella* agar and suspended in *Brucella* broth for 24 h before use in experiments. *H. pylori* bacteria were exposed in the presence or absence of test compounds as described previously [35], and detailed assay procedures can be found in the Supplementary Information.

### 4.5 Acid Stability of Test Compounds

The nitrofuran derivatives CLI‐19‐008, CLI‐19‐009, CLI‐03‐303, and CLI‐03‐258 were evaluated for stability under neutral and acidic conditions; detailed methodology can be found in the Supplementary Information.

### 4.6 RNA‐Sequencing

RNA‐sequencing was used to investigate the transcriptional responses of *H. pylori* to CLI‐19‐009; detailed methodology can be found in the Supplementary Information.

### 4.7 Checkerboard Assay

A series of checkerboard assays were conducted to evaluate the synergism between test compounds and clinical antibiotics or PPIs [36]. Fractional inhibitory concentration index (FICI) values were interpreted as follows: ≤ 0.5, synergistic interaction; > 0.5 to ≤ 1.0, additive interaction; > 1.0 to < 4.0, indifferent interaction; and ≥ 4.0, antagonistic interaction [37]. These predefined cutoffs were applied consistently throughout the study and the detailed methodology can be found in the Supplementary Information.

### 4.8 Membrane Potential and Depolarization Assay

The membrane potential assays [34] and membrane depolarization assays [38] were done as previously described. The detailed assay procedure can be found in the Supplementary Information.

### 4.9 Mammalian Cell Cytotoxicity Assay

The hepatic cell line HepG2, kidney cell line HKC, and two gastric cell lines, AGS and MKN‐28 [39], were used to determine cytotoxicity. The detailed assay procedure can be found in the Supplementary Information.

### 4.10 *Galleria mellonella*–*H. pylori* Infection Assay

Cost effective *G. mellonella* larvae (Vanderhorst Inc., St. Mary's, OH, USA) were used in this study as previously described [40]. The detailed assay procedure can be found in the Supplementary Information.

### 4.11 Animal Studies

C57BL/6J mice [41] were purchased from Charles River Laboratories (Bar Harbor, ME, USA). Detailed assay procedures can be found in the Supplementary Information.

### 4.12 Mutational Frequency Assay

Two lineages of *H. pylori* from the same clone were passaged continuously to evaluate mutation emergence against CLI‐19‐009. Serially diluted compounds were added to *H. pylori* and passaged two times a week; an aliquot of cells from every passage was stored frozen. After 33 days, the mutational frequency was recorded, and the frozen cells were cultured in *Brucella* agar with 10% FBS and sent to the Seq‐center (Microbial Sequencing Center, PA, USA) for whole‐genome sequencing to identify resistance mutations [34].

### 4.13 Statistical Analysis

Statistical analysis was performed using one‐ or two‐way ANOVA and two‐way analysis of variance followed by Bonferroni post‐test or Dunnett's test in GraphPad Prism version 10 (GraphPad Software, La Jolla CA, USA). A *p*‐value < 0.05 was considered statistically significant.

## Author Contributions

N.T. and E.M. conceived the in vitro and in vivo projects, designed the study, and led and supervised the work. N.T. performed the MIC assays, killing kinetics, fluorescence probe assays, checkerboard studies, scanning electron microscopy experiments, mammalian cell models for toxicity, and animal studies. N.T. performed the mutational frequency assay and selected the resistant mutants. K.A. conducted the bioinformatic analysis of RNA‐sequencing and generated the figures. S.L. and S.D. analyzed and scored the H&E‐stained gastric tissues. S.G., J.L., and G.J. designed, synthesized, validated, and provided the nitrofuran derivatives. All authors have read and approved the final manuscript.

## Ethics Statement

Animal studies were conducted under protocols approved by the Institutional Animal Care and Use Committee (IACUC). Approval for the study, *“*Therapeutic Compounds Against *Helicobacter pylori* in Mice at HMRI*,”* was obtained from HMRI (Approval No. IS00007516) and Rhode Island Hospital (CMTT No. 503619).

## Conflicts of Interest

This study was an independent submission. After this study was submitted, author, Eleftherios Mylonakis was invited to serve as an Editorial Board Member of MedComm. Author Eleftherios Mylonakis is an Editorial board member of MedComm. Eleftherios Mylonakis was not involved in the journal's review of or decisions related to this manuscript. Scott Goodrich, Joseph St. Laurent, and Gerald Jones are officers and/or shareholders of KODA Therapeutics Inc., as well as the original inventors of the nitrofuran derivatives described in this study. KODA Therapeutics Inc. is the owner of the patent family inclusive of US Patent No. 12,369,579, which covers the compounds reported here. Scott Goodrich, Joseph St. Laurent, and Gerald Jones are the named inventors of this patent family and contributed to the synthesis of the compounds used in this study but had no role in the biological investigations, data analysis, or manuscript preparation, except for proofreading and approving the final version for publication. The other authors declare no conflicts of interest.

## Supporting information




**Supporting file 1**: mco271057‐sup‐0001‐SuppMat.pdf

## Data Availability

The RNA‐seq datasets generated and/or analyzed during the current study have been deposited in the [Zenodo.org] repository under the https://doi.org/10.5281/zenodo.15498141.
