# Supplementary material for: Acid‐Stable Nitrofuran Triple Therapy With Rabeprazole and Amoxicillin Targeting Helicobacter pylori Infection
Source: MedComm (2020). 2026 Oct 11;7(10):e71057. doi: 10.1002/mco2.71057 (PMC13655363; doi:10.1002/mco2.71057)
Supplement: Supplementary file 1 [file MCO2-7-e71057-s001.pdf]

Acid-Stable Nitrofuran Triple Therapy with Rabeprazole and Amoxicillin Targeting *Helicobacter pylori* Infection

Nagendran Tharmalingam[1,2,*], Kulandaisamy Arulsamy[3], Shaolei Lu[4], Sadhna Dhingra[5], Scott Goodrich[6], Joseph St Laurent[6], Gerald Jones[6], Eleftherios Mylonakis[1,2,*]

[1]Department of Medicine, Houston Methodist Research Institute, Houston, TX, USA

[2]Department of Medicine, Weill Cornell Medicine, New York, NY, USA.

[3]Boston Children's Hospital, Boston, MA, USA.

[4]Department of Pathology, Rhode Island Hospital, Warren Alpert Medical School of Brown University, Providence, RI, USA.

[5]Department of Pathology, Houston Methodist Hospital, Houston, TX, USA

[6]Koda Therapeutics, West Bridgewater, MA, USA.

Corresponding author: emylonakis@houstonmethodist.org

Co-corresponding author: ntharmalingam@houstonmethodist.org

Supplementary methods.

Synthesis of 5-nitro-2-furaldehyde N-(ethyl phenylphosphoramidoyl)hydrazone (CLI-03-258). A solution of aniline (5.7 g, 60 mmol) in dichloromethane (DCM; 1 mL/mmol) triethylamine (TEA, 1 equivalent) was added dropwise to an equimolar solution of ethyl dichlorophosphate in DCM stirred at room temperature (RT). After ~18 hours, the reaction mixture was concentrated, and the residue was treated with tetrahydrofuran (THF; 1 mL/mmol) and vacuum-filtered to remove TEA HCl. The filtrate was added dropwise to a mixture of hydrazine hydrate (4 equivalents) in an equal volume of THF stirred at 0°C. Stirring was continued overnight at RT, after which the lower phase of the reaction mixture was discarded, and the THF solution was concentrated. Crude ethyl phosphoranilidohydrazide was triturated with ether, collected by vacuum filtration, washed with ether, air-dried, and used directly in the next step without further purification.

A mixture of the crude hydrazide and equimolar 5-nitro-2-furaldehyde in methanol (5 mL/mmol hydrazide) was stirred at reflux for 3 hours. After cooling to RT, the reaction mixture was vacuum-filtered, and the filtrate was concentrated under reduced pressure. The crude hydrazone was crystallized from hot aqueous acetonitrile (ACN; 1:4; 5 mL/g): 10.94 g [53% yield (based on aniline)]; yellow solid; m.p. 132-137°C; ^{1}H NMR (400 MHz, DMSO-d_6) d 10.224 (d, 1H, NH), 8.205 (d, 1H; NH), 7.880 (s, 1H, CH=N), 7.719 (d, *J*=4 Hz, 1H, furan), 7.193 (t, 2H, phenyl), 7.109 (m, 2H, phenyl), 6.994 (d, *J*=4 Hz, 1H, furan), 6.852 (m, 1H, phenyl), 4.103 (m, 2H, CH_2), 1.294 (t, 3H, CH_3); HPLC-DAD-MS: 98.7% (200 nm); *m/z* 339.0 $[M+H]^+$; 337.0 $[M-H]^-$; Calcd for $C_{13}H_{15}N_4O_5P$•0.5 H_2O (347.26): C, 44.96; H, 4.64; N, 16.13. Found: C, 44.87; H, 4.63; N,16.04.

Synthesis of 6,7-dihydro-5H-nitrofuran-4-one. A solution of 1,3-cyclohexanedione (169.45 g, 1.5 mol) in water (10 mL/g) containing THF (1 mL/g) was added dropwise to a mixture of chloroacetaldehyde (1.25 equivalents) in water (4 mL/mL chloroacetaldehyde) containing sodium bicarbonate (1.25 equivalents) stirred at 0°C. After stirring overnight at RT, the pH was adjusted to ~1 with concentrated sulfuric acid, and the mixture was extracted with ethyl acetate. The aqueous phase was extracted with ethyl acetate, and the combined extracts were washed with brine, dried ($MgSO_4$), filtered, and concentrated to give 6,7-dihydro-5H-nitrofuran-4-one as a white solid: 124 g (61%).

Synthesis of 2-nitro-6,7-dihydro-5H-benzofuran-4-one. 6,7-dihydro-5H-nitrofuran-4-one (124 g, 900 mmol) was nitrated by the careful addition of sodium nitrate (1 equivalent) to cold concentrated sulfuric acid (1 mL/mmol substrate). The reaction mixture was poured over ice, and the crude product was isolated by vacuum filtration, washed extensively with water, and purified by flash chromatography to give 2-nitro-6,7-dihydro-5H-benzofuran-4-one as a golden-yellow crystalline solid: 87.04 g (53%); 99.5+% [UHPLC (210 nm)].

Synthesis of 2-nitro-6,7-dihydrobenzofuran-4-one semicarbazone (CLI-03-303). An equimolar mixture of semicarbazide HCl and 2-nitro-6,7-dihydro-5H-nitrofuran-4-one in 95% ethanol (12 mL/mmol) was heated at 80°C for 1 hour. The product precipitated from the cooled reaction mixture was collected by vacuum filtration, washed with 95% ethanol, and air-dried to give 2-nitro-6,7-dihydronitrofuran-4-one semicarbazone as a bright yellow solid.

Synthesis of 2-nitro-6,7-dihydrobenzo**furan-4-one N-(ethyl phenylphosphoramidoyl)hydrazone (CLI-19-008).** An equimolar mixture of ethyl phosphoranilidohydrazide and 2-nitro-6,7-dihydro-5H-benzofuran-4-one in methanol (3 mL/mmol) was heated at 70°C for 3 hours. The reaction mixture was concentrated, and the residue was triturated with water, collected by vacuum filtration, and purified by flash chromatography.

Synthesis of 2-nitro-6,7-dihydrobenzofuran-4-one N-(cinnamoyl)hydrazone (CLI-19-009). An equimolar mixture of cinnamic acid hydrazide and 2-nitro-6,7-dihydro-5H-benzofuran-4-one in methanol (5 mL/mmol) was heated at 70°C for 4 hours. After cooling, the pure product was collected by vacuum filtration, washed with cold methanol, and air-dried.

Chromatographic assay. Approximately 0.1 g of each analyte was transferred into a 100-mL volumetric flask, and 50 mL of DMSO was added to create a primary stock solution of CLI-09-009, CLI-19-008, CLI-258, and CLI-003. Mechanical means were used to ensure the complete dissolution of the test materials. Each flask was then QS (e.g., to 100 mL) with methanol, yielding a final concentration of approximately 1 mg/mL of each analyte. Subsequently, a quantitation curve from 2.5 to 250 μg/mL of each target analyte was prepared via quantitative serial dilution into the diluent (e.g., 50/50 DMSO/methanol). Each reference standard was assayed in triplicate using an Agilent Model 1200 UPLC equipped with an Agilent, Zorbax

C-18, 4.6 X 250 mm, 5 µm analysis column, and Agilent diode array detector cable of data collection at 210, 254, 280, and 305 nm. Quantitative analysis was completed as needed, and data fitted to the least-squares fit curve and solution concentrations were confirmed using a suitable Relative Response Factor determination.

Acid stability of test compounds. The nitrofuran derivatives CLI-19-008, CLI-19-009, CLI-03-303, and CLI-03-258 were evaluated for stability under neutral and acidic conditions. Each compound was analyzed in its untreated form and following exposure to pH 2, yielding a total of eight samples.

Sample Preparation: All four nitrofuran derivatives (CLI-19-008, CLI-19-009, CLI-03-303, and CLI-03-258) were stored as 10 mg/mL stock solutions. The pH of acetonitrile was adjusted to 2.0 and subsequently used to prepare the stock solutions. The solutions were incubated at room temperature for 24 h and then used to evaluate acid stability. Nitrofuran compounds dissolved in non-acidified acetonitrile were used as controls. The stock solutions were analyzed at the Mass Spectrometry Facility (MSF) at the The University of Texas Medical Branch. Briefly, 20 µL of each sample was transferred into a 1.5 mL microcentrifuge tube, and the compounds were dried under a gentle stream of nitrogen gas. The resulting residues were reconstituted in 80% (v/v) acetonitrile to achieve a concentration of 1 mg/mL. The samples were subsequently diluted to a final concentration of 100 µg/mL using the same solvent system and vortexed to ensure complete dissolution.

Flow-Injection Analysis–Mass Spectrometry (FIA–MS): Mass spectrometric analyses were performed using a Vanquish liquid chromatography system coupled to a Fusion Tribrid Orbitrap mass spectrometer (Thermo Scientific) [66]. For FIA-MS analysis, 20 µL of each prepared sample was injected into a carrier stream consisting of 70% (v/v) acetonitrile at a flow rate of 150 µL/min. Data acquisition was conducted in positive electrospray ionization mode with a resolving power of 120,000 (at m/z 200). Under these conditions, analytes were predominantly detected as protonated molecular ions ([M+H]+). Page no- 23-24, Line no-533-543.

Data Acquisition and Processing: Raw mass spectral data were acquired using instrument control software and subsequently exported from Freestyle (v1.8.65.0, Thermo Scientific) in tabulated format.

Data analysis was performed using custom scripts written in R (v4.4.3) within the RStudio environment (v2026.04.0). Data preprocessing, manipulation, and visualization were conducted using the packages data.table (v1.18.2), ggplot2 (v4.0.3), and tidyverse (v2.0.0). Spectral profiles were compared between untreated and pH 2-treated samples to assess changes in mass distribution, signal intensity, and the presence of potential degradation or transformation products.

Comparative Analysis: For each compound, mass spectra were obtained under neutral and acidic conditions. Differences in the relative abundance of the [M+H]+ ions and the emergence of additional peaks were interpreted as indicators of compound stability or chemical modification under low pH conditions.

RNA- sequencing.

Transcriptional responses in H. pylori to CLI-19-009 using RNA-seq. *H. pylori* 60190 used in this assay. *H. pylori* was grown in MHB+10% FBS in the presence or absence of sub-MIC of CLI-19-009 with agitation for 5 h. The cells were washed and treated with Trizol reagent and sent to LCSciences (Houston, TX, USA). Raw sequencing reads were preprocessed using Trim Galore[68] with default parameters to remove adapter sequences and low-quality bases. High-quality reads were subsequently aligned to the *H. pylori* reference genome (NCBI RefSeq assembly GCF_017821535.1) using the STAR aligner[69] together with the corresponding genome annotation in GTF format. Gene-level read counts were quantified using featureCounts[70] and used as input for downstream differential expression analysis.

Differential gene expression analysis was performed in R using DESeq2[71]. Genes with fewer than 10 counts in at least three samples were excluded prior to analysis. Raw count matrices were normalized using DESeq2 size-factor normalization based on the median-of-ratios method. Differential expression between CLI-19-009-treated and control samples was assessed using the Wald test with the contrast defined as CLI-19-009 versus control. Multiple testing correction was performed using the Benjamini–Hochberg false discovery rate (FDR) method. Genes with FDR-adjusted P values < 0.05 were considered significantly differentially expressed and these

genes were visualized by volcano plot. Log2 fold-change shrinkage was performed using the apeglm method implemented in DESeq2 for visualization and effect-size stabilization. Pearson correlation analysis was performed using variance-stabilized expression values to assess transcriptomic reproducibility and global sample relationships between control and CLI-19-009-treated samples.

Pathway enrichment analysis

For functional interpretation, protein identifiers were annotated using BlastKOALA[72]-based KEGG Orthology (KO) assignments. KO identifiers were mapped to KEGG pathways to generate custom TERM2GENE and TERM2NAME annotation databases for enrichment analyses. Over-representation analysis (ORA) was performed using the enricher function implemented in clusterProfiler[73] with significantly upregulated and downregulated genes analyzed separately. Statistical significance of pathway enrichment was assessed using P values < 0.05.

*RNA sequencing of gastric epithelial cells***.** MKN-28 gastric epithelial cells were seeded in 6 well plates for 24h. The cells were washed with antibiotic and serum free DMEM and infected with *H. pylori* (MOI 200) for 2 hours to promote infection and host–pathogen interactions. Following this infection period, non-adherent bacteria were removed by washing with sterile PBS, after which CLI-19-009 (4xMIC) added and cells were incubated for an additional 22 hours prior to RNA-seq analysis. The treated cells were suspended in Trizol reagent, and the ribonucleic acid (RNA) was isolated [7]. The isolated RNA was then sent to LCSciences (Houston, TX, USA) for sequencing. The raw FASTQ files of RNA sequencing were processed and aligned against the human-reference genome by the STAR [69] aligner program. Next BAM (Binary Alignment Map) files were used for gene read quantification with feature count [70]. Differnetial gene expression analysis was performed using the DESeq Rpackage [71], which identifies significantly upregulated and downregulated genes from RNA-seq count data, followed by gene pathway enrichment analysis using the clusterProfiler bioinformatics package to identify biologically relevant pathways associated with the transcriptional changes.

Supplementary Information

A

Agents	IC50		Anti-*H. pylori* activity	
	HepG2 (mg/L)	HKC (mg/L)	MIC	MBC
1	16	32	2	2
2	>64	>64	2	2
3	16	32	1	2
4	32	32	1	2
5	>64	>64	2	2
6	>64	>64	2	2
7	>64	>64	4	4
8	4	4	4	4
9	>64	>64	4	4
10	>64	>64	1	1
11	>64	>64	1	1
12- CLI-03-303	>**64**	>**64**	**0.25**	**0.25**
13	>64	>64	4	8
14- CLI-19-008	>**64**	>**64**	**0.25**	**0.25**
15- CLI-19-009	>**64**	>**64**	**0.25**	**0.25**
CLI 258	>64	>64	1	2
Amoxicillin	>64	>64	0.01	0.01
Clarithromycin	>64	>64	0.001	0.001

B

Strains	CLI-19-008	CLI-19-008	CLI-03-303	CLI-03-258	Amox	Clar	Met	Nit
2685	0.25	0.03	0.125	0.5	0.03	0.01	0.25	0.5
2715	0.125	0.03	0.125	0.5	0.03	0.03	2	0.25
3427	0.25	0.5	1	1	0.01	>0.5	2	0.25
2689	0.25	0.25	0.25	1	0.06	0.06	0.5	0.25
2698	0.25	0.06	0.25	1	0.06	0.03	0.5	0.25
2687	0.25	0.06	0.25	1	0.06	0.03	0.5	1
2727	0.5	0.25	0.5	0.5	0.01	0.005	2	0.25
3412	0.5	0.06	0.25	1	0.06	0.01	0.5	0.25
3413	0.25	0.03	0.125	1	0.03	0.01	0.5	0.25
3424	4	0.5	1	2	0.06	>4	4	0.5
ATCC 43579	0.5	0.06	0.25	0.5	0.06	0.001	1	0.5

C

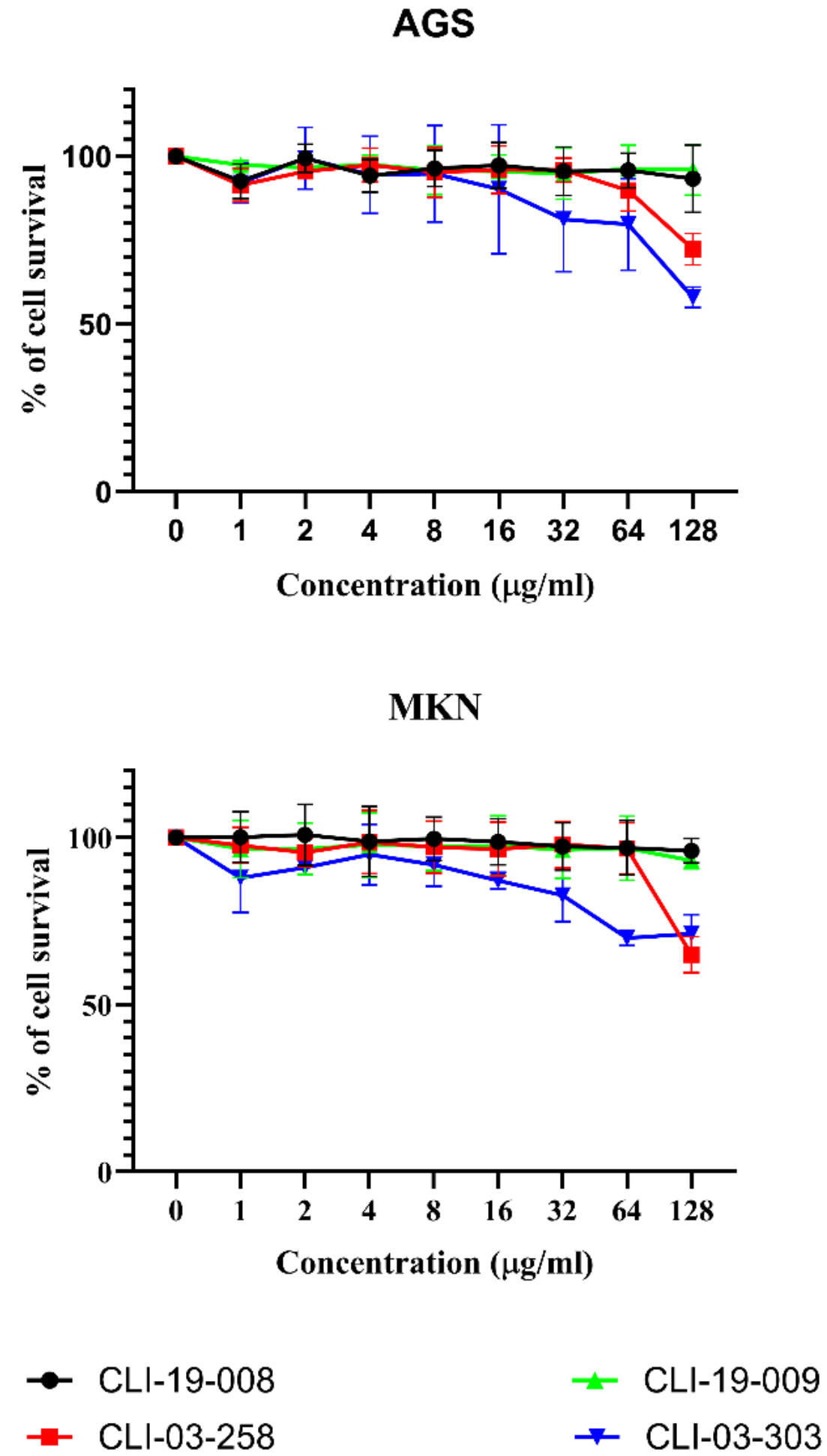

Figure S1. MICs and Cytotoxicity of analogs. A. MICs of analogs against *H. pylori* 60190 and IC_{50}. Broth microdilution assays were carried out to evaluate the anti-*H. pylori* activity. **B. Antibacterial activity of nitrofuran derivatives against clinical *H. pylori* isolates**. MICs (mg/L) of nitrofuran derivatives (CLI-19-008, CLI-19-009, CLI-03-303, CLI-03-258) were determined against clinical *H. pylori* isolates with variable susceptibility and reference strain ATCC 43579. Amoxicillin (Amox), clarithromycin (Clar), metronidazole (Met), and nitrofurantoin (Nit) were included for comparison. CLI-19-009 showed consistent activity across isolates, including those with reduced susceptibility to clarithromycin and metronidazole. “>” indicates no inhibition at highest concentration tested. **C. *In vitro* cytotoxicity**. Cytotoxicity of four selected nitrofuran derivatives was assessed against gastric cell lines. CLI-19-008, CLI-19-009, CLI-03-303, CLI-03-258 has an IC50 > 64 mg/L and each experiment was performed in triplicate, and untreated cells were used as controls for normalization.

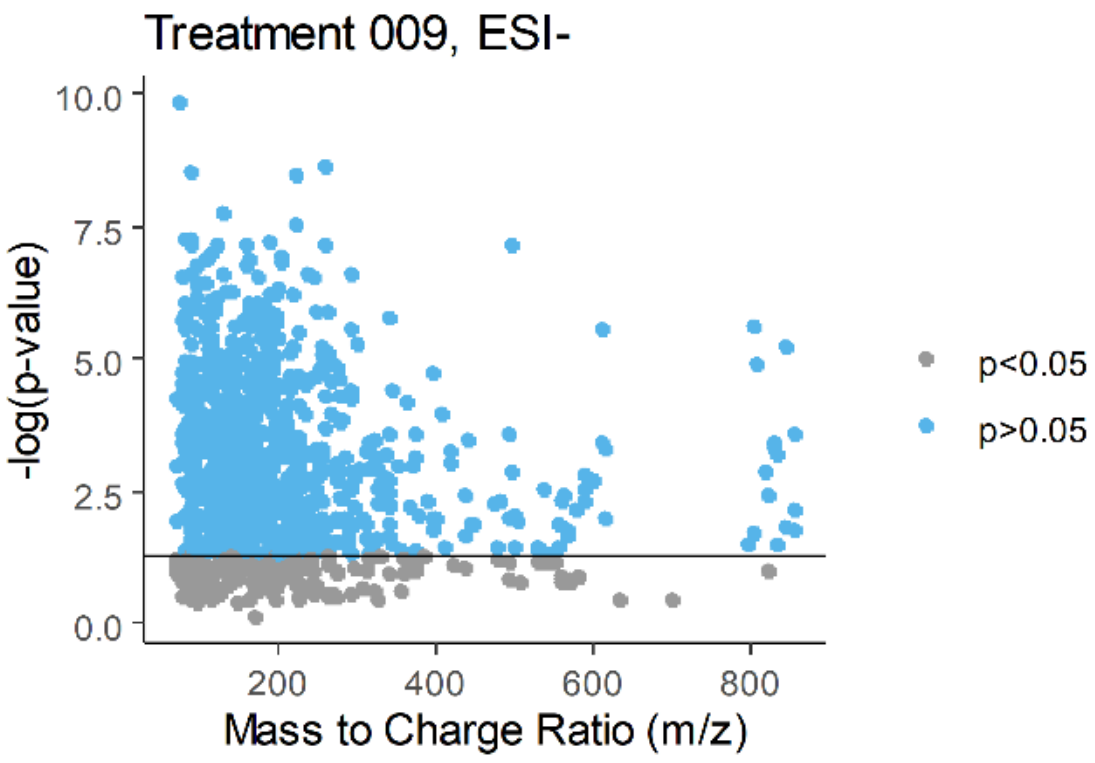
Treatment 009, ESI-
10.0
7.5
5.0
2.5
0.0
-log(p-value)
200
400
600
800
Mass to Charge Ratio (m/z)
p<0.05
p>0.05

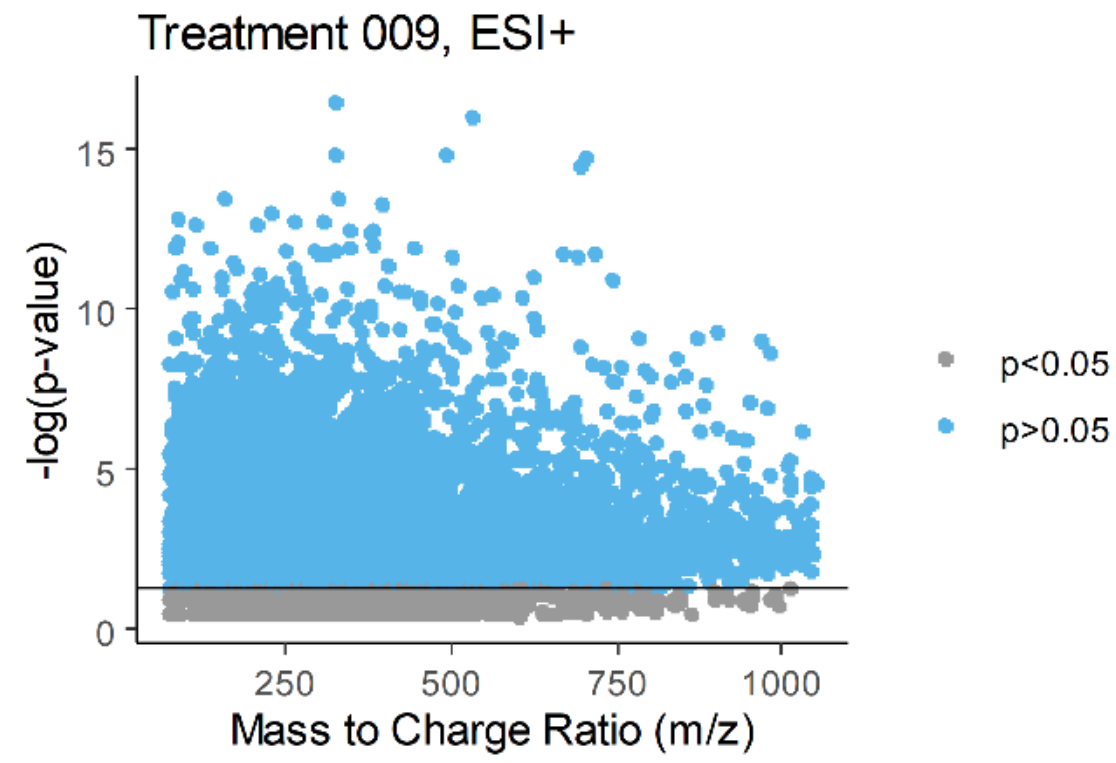
Treatment 009, ESI+
15
10
5
0
-log(p-value)
250
500
750
1000
Mass to Charge Ratio (m/z)
p<0.05
p>0.05

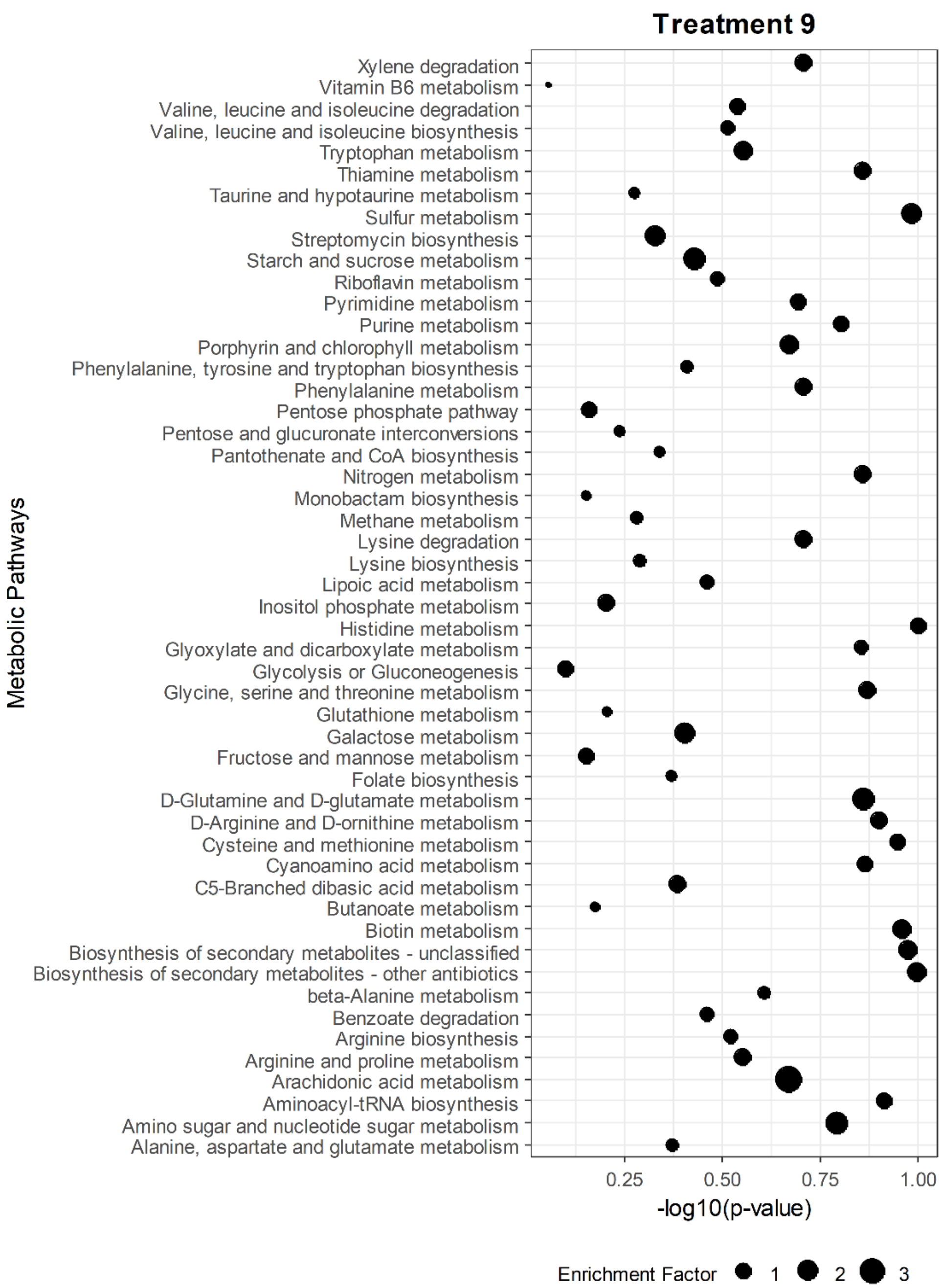
Treatment 9
Metabolic Pathways
Xylene degradation
Vitamin B6 metabolism
Valine, leucine and isoleucine degradation
Valine, leucine and isoleucine biosynthesis
Tryptophan metabolism
Thiamine metabolism
Taurine and hypotaurine metabolism
Sulfur metabolism
Streptomycin biosynthesis
Starch and sucrose metabolism
Riboflavin metabolism
Pyrimidine metabolism
Purine metabolism
Porphyrin and chlorophyll metabolism
Phenylalanine, tyrosine and tryptophan biosynthesis
Phenylalanine metabolism
Pentose phosphate pathway
Pentose and glucuronate interconversions
Pantothenate and CoA biosynthesis
Nitrogen metabolism
Monobactam biosynthesis
Methane metabolism
Lysine degradation
Lysine biosynthesis
Lipoic acid metabolism
Inositol phosphate metabolism
Histidine metabolism
Glyoxylate and dicarboxylate metabolism
Glycolysis or Gluconeogenesis
Glycine, serine and threonine metabolism
Glutathione metabolism
Galactose metabolism
Fructose and mannose metabolism
Folate biosynthesis
D-Glutamine and D-glutamate metabolism
D-Arginine and D-ornithine metabolism
Cysteine and methionine metabolism
Cyanoamino acid metabolism
C5-Branched dibasic acid metabolism
Butanoate metabolism
Biotin metabolism
Biosynthesis of secondary metabolites - unclassified
Biosynthesis of secondary metabolites - other antibiotics
beta-Alanine metabolism
Benzoate degradation
Arginine biosynthesis
Arginine and proline metabolism
Arachidonic acid metabolism
Aminoacyl-tRNA biosynthesis
Amino sugar and nucleotide sugar metabolism
Alanine, aspartate and glutamate metabolism
0.25
0.50
0.75
1.00
-log10(p-value)
Enrichment Factor
1
2
3

Figure S2. Untargetted metabolomics of CLI-19-009 against *H. pylori*. *H. pylori* was treated with CLI-19-009 and frozen cells were submitted to Brown University Mass-Spec core for untargeted metabolomics. CLI-19-009 show it intervened aminoacid, nucleotide and nitrogen metabolism.

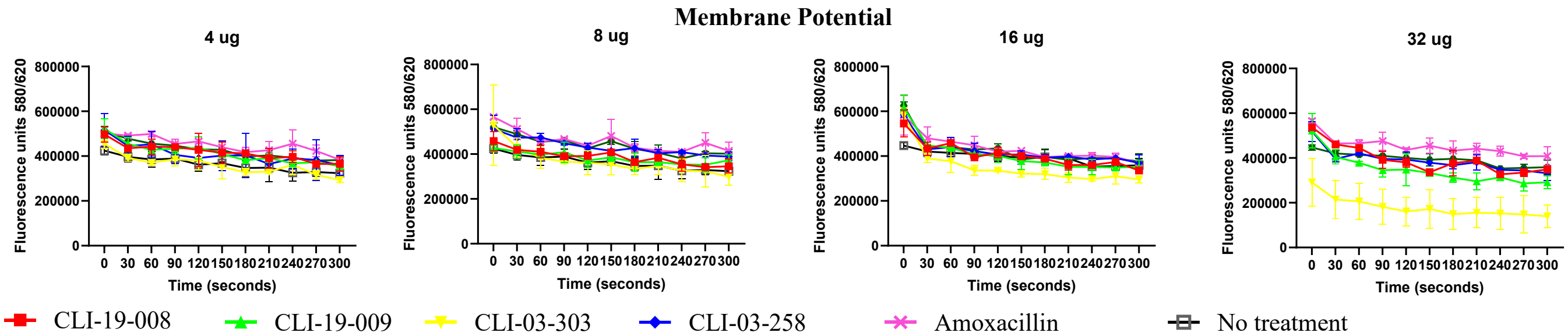
Membrane Potential
4 ug
8 ug
16 ug
32 ug
Fluorescence units 580/620
Time (seconds)
CLI-19-008
CLI-19-009
CLI-03-303
CLI-03-258
Amoxacillin
No treatment

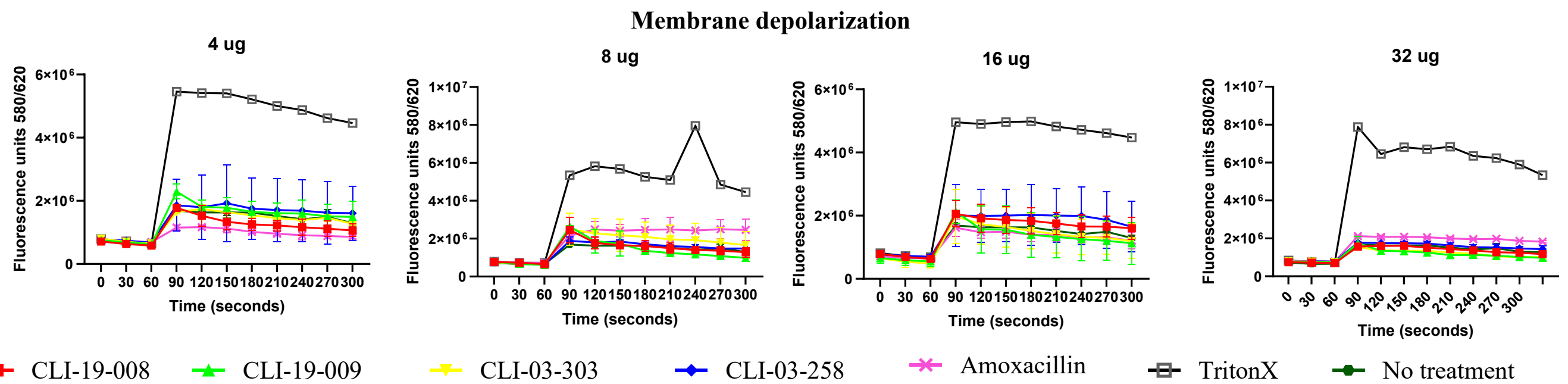
Membrane depolarization
4 ug
8 ug
16 ug
32 ug
Fluorescence units 580/620
Time (seconds)
CLI-19-008
CLI-19-009
CLI-03-303
CLI-03-258
Amoxacillin
TritonX
No treatment

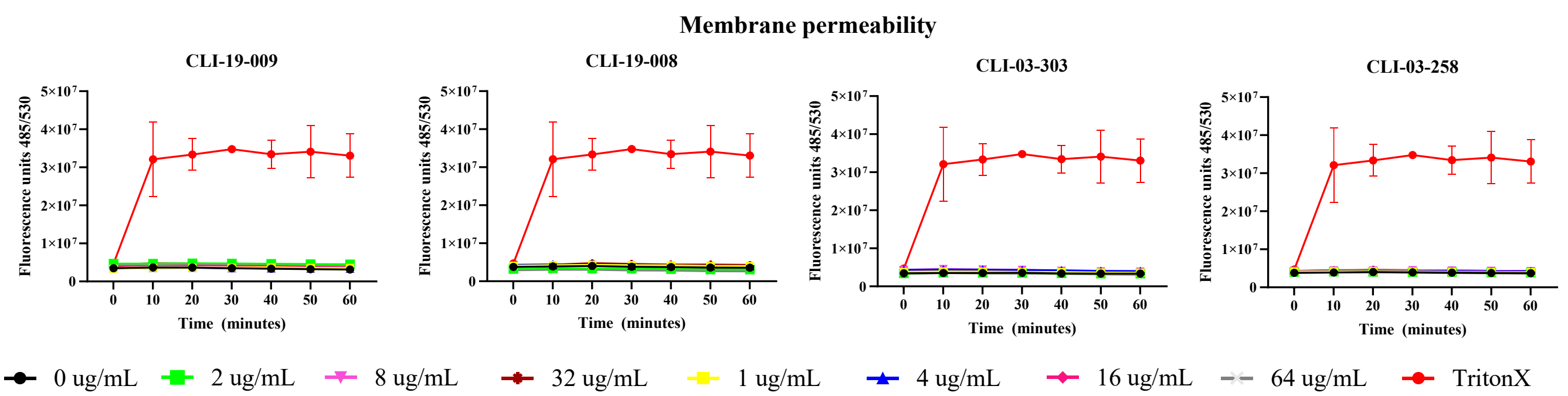
Membrane permeability
CLI-19-009
CLI-19-008
CLI-03-303
CLI-03-258
Fluorescence units 485/530
Time (minutes)
0 ug/mL
2 ug/mL
8 ug/mL
32 ug/mL
1 ug/mL
4 ug/mL
16 ug/mL
64 ug/mL
TritonX

Figure S3. Membrane activity of nitrofuran derivatives against *H. pylori.* Cells were treated with fluorescent probes such as DISC3(5) for membrane potential and depolarization studies, and Sytox Green for membrane permeability analysis, in the presence or absence of test compounds. The experiments demonstrated that the test compounds did not cause any significant cellular fluorescence show the bacterial inhibition is independent of membrane targeting. Triton-X and CCCP were used as positive controls for membrane disruption and depolarization, respectively. All assays were performed in triplicate, and fluorescence data were normalized against untreated controls to ensure accuracy.

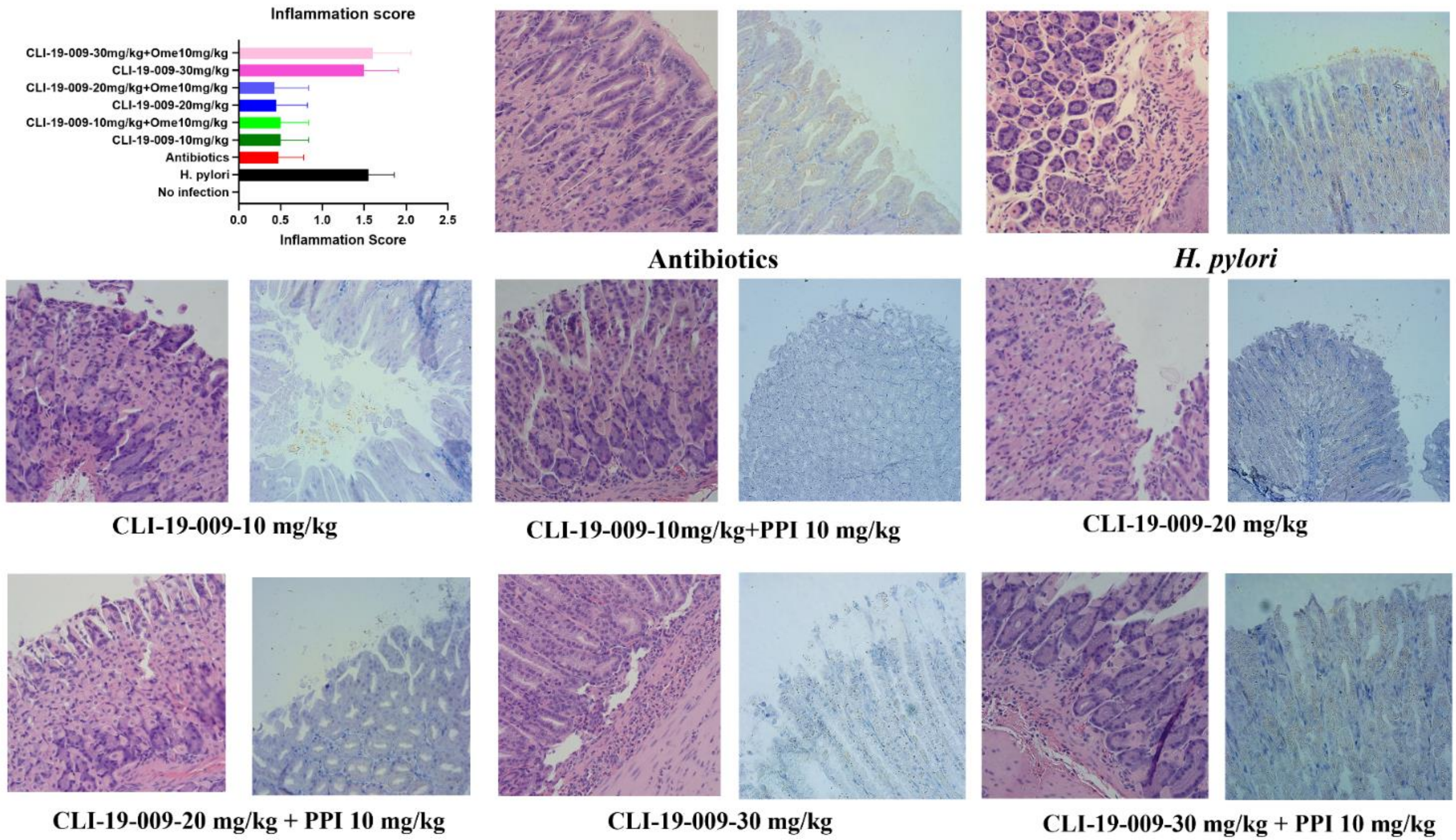
Inflammation score
CLI-19-009-30mg/kg+Ome10mg/kg
CLI-19-009-30mg/kg
CLI-19-009-20mg/kg+Ome10mg/kg
CLI-19-009-20mg/kg
CLI-19-009-10mg/kg+Ome10mg/kg
CLI-19-009-10mg/kg
Antibiotics
H. pylori
No infection
0.0 0.5 1.0 1.5 2.0 2.5
Inflammation Score
Antibiotics
H. pylori
CLI-19-009-10 mg/kg
CLI-19-009-10mg/kg+PPI 10 mg/kg
CLI-19-009-20 mg/kg
CLI-19-009-20 mg/kg + PPI 10 mg/kg
CLI-19-009-30 mg/kg
CLI-19-009-30 mg/kg + PPI 10 mg/kg

Figure S4. *In vivo* efficacy of CLI-19-009 against *H. pylori* infection in dual therapy. A. Hematoxylin and eosin (H&E)-stained stomach sections from *H. pylori*-infected C57BL/6J mice were analyzed for inflammation (n=10). Inflammatory cells, including neutrophils and lymphocytes, were quantified in each section. *H. pylori*-infected stomach sections exhibited a high number of inflammatory cells. CLI-19-009 at 10 mg/kg with omeprazole had an inflammation score of 0.5 to 0.75 compared to untreated control. **B**. Representative images of stomach sections from experimental groups stained with H&E and polyclonal *H. pylori* antibody to visualize bacterial colonization and inflammatory response are shown. Representative images of the stained stomach sections.

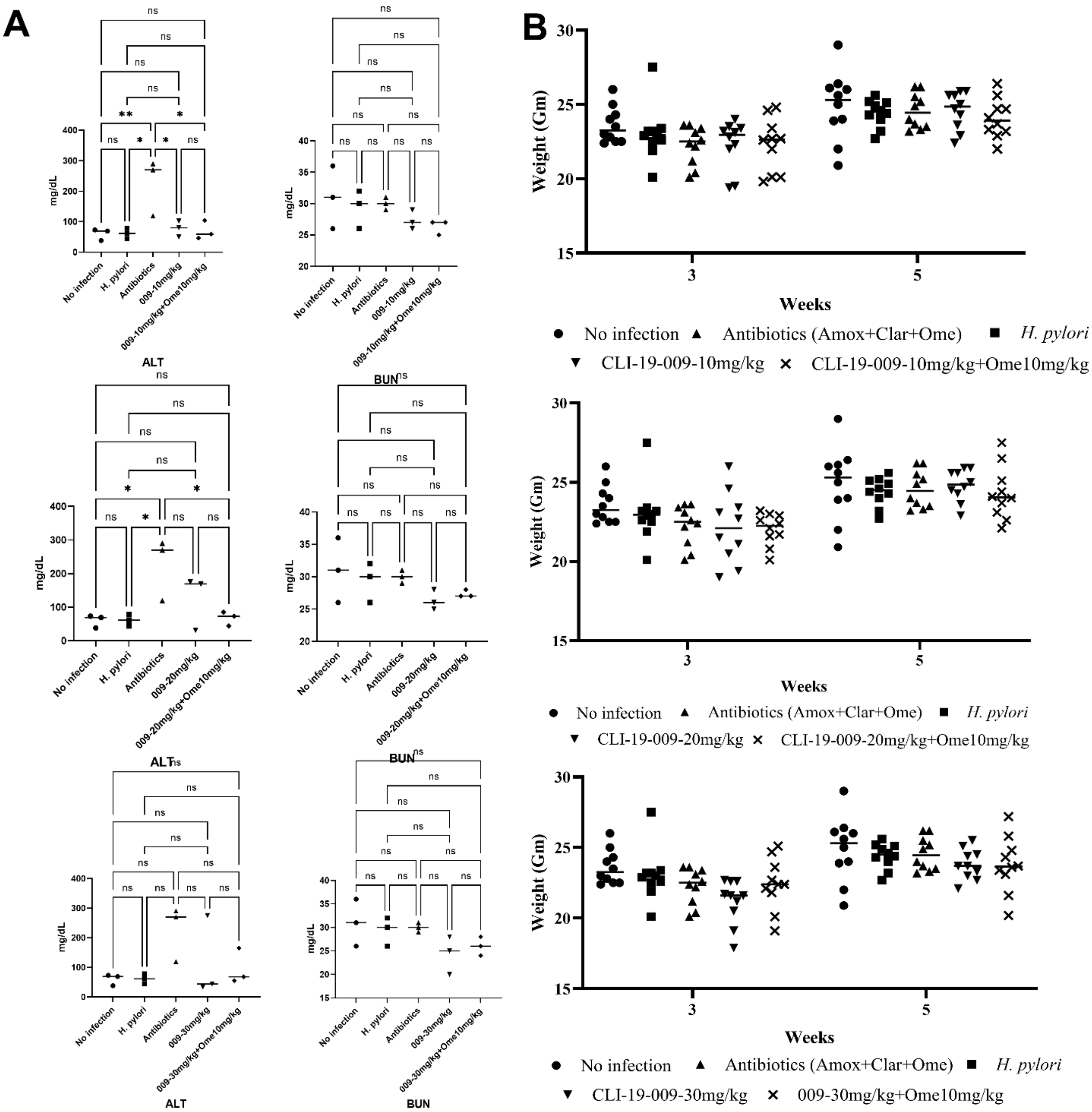

A
B
mg/dL
No infection
H. pylori
Antibiotics
009-10mg/kg
009-10mg/kg+Ome10mg/kg
009-20mg/kg
009-20mg/kg+Ome10mg/kg
009-30mg/kg
009-30mg/kg+Ome10mg/kg
ALT
BUN
ns
*
**
Weight (Gm)
Weeks
No infection
Antibiotics (Amox+Clar+Ome)
H. pylori
CLI-19-009-10mg/kg
CLI-19-009-10mg/kg+Ome10mg/kg
CLI-19-009-20mg/kg
CLI-19-009-20mg/kg+Ome10mg/kg
CLI-19-009-30mg/kg
009-30mg/kg+Ome10mg/kg

Figure S5. ***In vivo*** **toxicity, and weight change in dual therapy. A**. Whole blood samples were collected from mice treated with CLI-19-009 either in the presence or absence of a PPI and sent to a clinical laboratory for the estimation of alanine aminotransferase (ALT) and blood urea nitrogen (BUN) levels, which serve as markers for liver and kidney function, respectively. Values are lesser than control antibiotics therapy group. **B**. Analysis of body weights measured before and after oral drug therapy show no significant changes between groups.

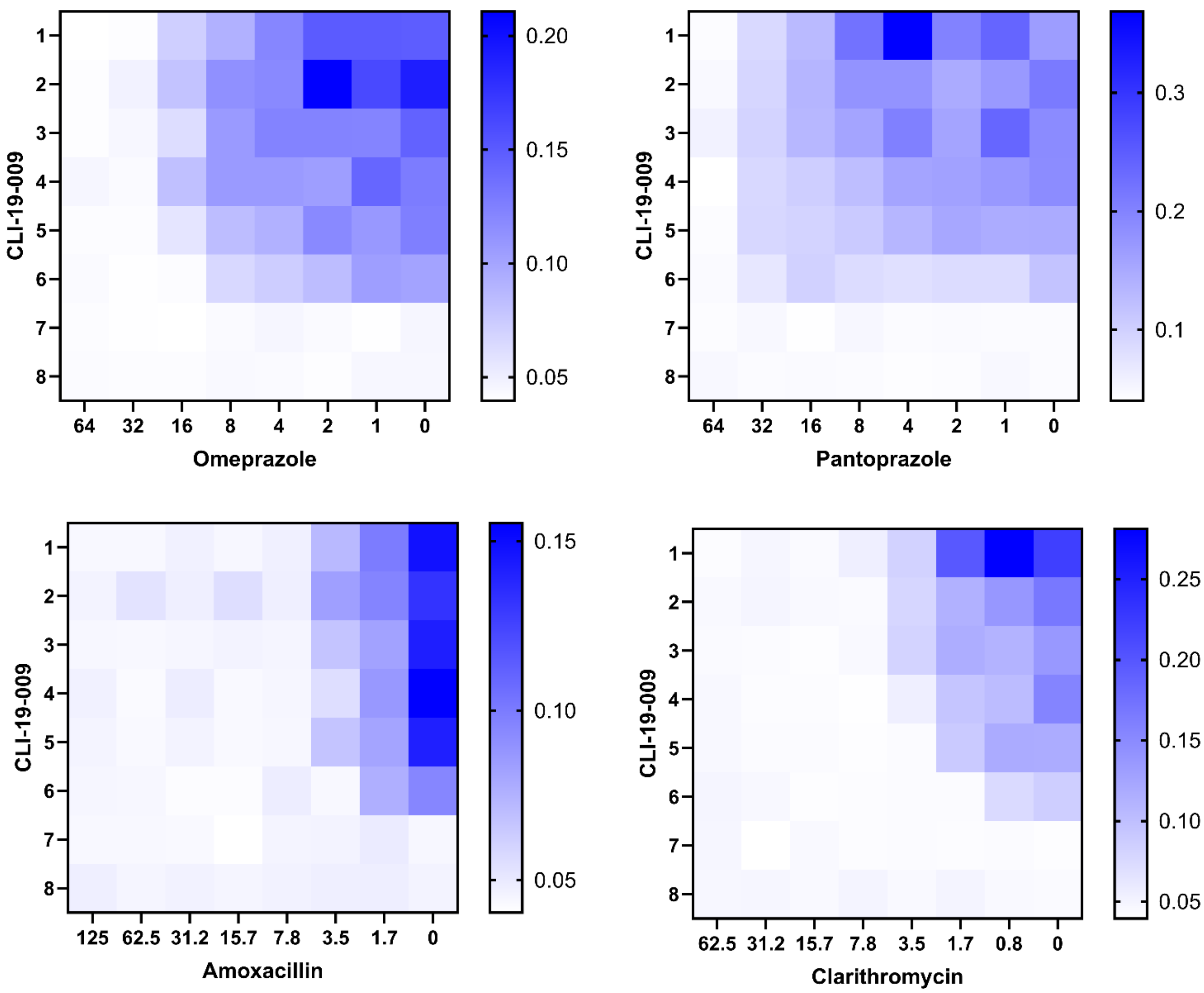
CLI-19-009
1
2
3
4
5
6
7
8
64 32 16 8 4 2 1 0
Omeprazole
0.20
0.15
0.10
0.05
CLI-19-009
64 32 16 8 4 2 1 0
Pantoprazole
0.3
0.2
0.1
CLI-19-009
125 62.5 31.2 15.7 7.8 3.5 1.7 0
Amoxacillin
0.15
0.10
0.05
CLI-19-009
62.5 31.2 15.7 7.8 3.5 1.7 0.8 0
Clarithromycin
0.25
0.20
0.15
0.10
0.05

Figure S6. Checkerboard assay of CLI-19-009 with clinical drugs. Checkerboard assays show CLI-19-009 exhibited an additive effect with omeprazole, clarithromycin, or amoxicillin (FICI ranging from 0.75-1.0).

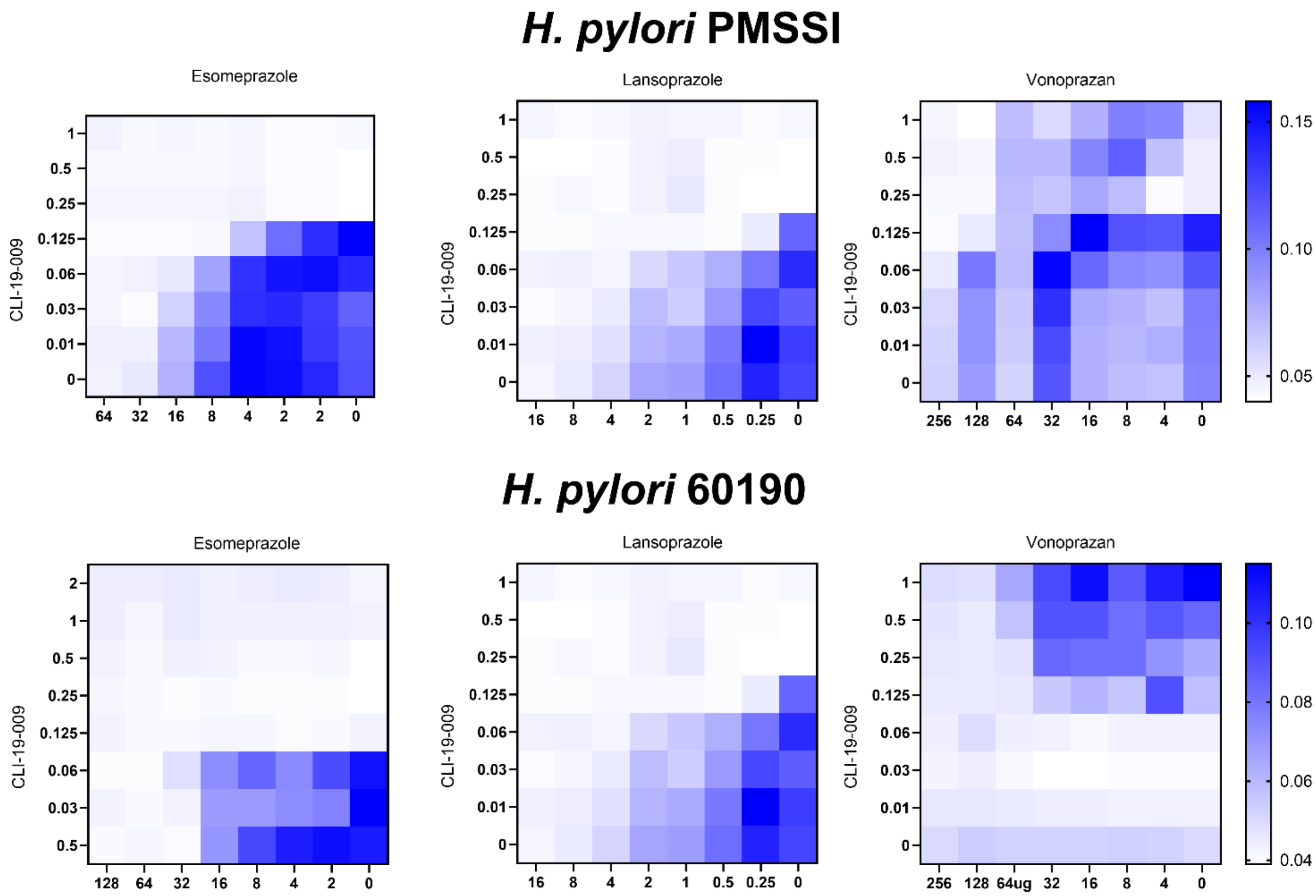
H. pylori PMSSI
Esomeprazole
Lansoprazole
Vonoprazan
CLI-19-009
1
0.5
0.25
0.125
0.06
0.03
0.01
0
64 32 16 8 4 2 2 0
16 8 4 2 1 0.5 0.25 0
256 128 64 32 16 8 4 0
0.15
0.10
0.05
H. pylori 60190
Esomeprazole
Lansoprazole
Vonoprazan
CLI-19-009
2
1
0.5
0.25
0.125
0.06
0.03
0.5
128 64 32 16 8 4 2 0
16 8 4 2 1 0.5 0.25 0
256 128 64ug 32 16 8 4 0
0.10
0.08
0.06
0.04

Figure S7. Checkerboard assay of CLI-19-009 with clinical PPIs against *H. pylori*. CLI-19-009 was tested in combination with clinical PPIs, including esomeprazole, lansoprazole, and vonoprazan and show addictive activity.
